# Distinct metabolic states of a cell guide alternate fates of mutational buffering through altered proteostasis

**DOI:** 10.1038/s41467-020-16804-6

**Published:** 2020-06-10

**Authors:** Kanika Verma, Kanika Saxena, Rajashekar Donaka, Aseem Chaphalkar, Manish Kumar Rai, Anurag Shukla, Zainab Zaidi, Rohan Dandage, Dhanasekaran Shanmugam, Kausik Chakraborty

**Affiliations:** 1 CSIR-Institute of Genomics and Integrative Biology, Mathura Road, Delhi, 110025 India; 2 Academy of Scientific and Innovative Research, CSIR-HRDC, Ghaziabad, Uttar Pradesh 201002 India; 3 CSIR-National Chemical Laboratory, Pashan Road, Pune, India; 4 Present Address: Institute of Biomedicine, University of Gothenburg, Medicinaregatan 7A, Gothenburg, Sweden

**Keywords:** Chaperones, Protein folding

## Abstract

Metabolic changes alter the cellular milieu; can this also change intracellular protein folding? Since proteostasis can modulate mutational buffering, if change in metabolism has the ability to change protein folding, arguably, it should also alter mutational buffering. Here we find that altered cellular metabolic states in *E. coli* buffer distinct mutations on model proteins. Buffered-mutants have folding problems in vivo and are differently chaperoned in different metabolic states. Notably, this assistance is dependent upon the metabolites and not on the increase in canonical chaperone machineries. Being able to reconstitute the folding assistance afforded by metabolites in vitro, we propose that changes in metabolite concentrations have the potential to alter protein folding capacity. Collectively, we unravel that the metabolite pools are bona fide members of proteostasis and aid in mutational buffering. Given the plasticity in cellular metabolism, we posit that metabolic alterations may play an important role in cellular proteostasis.

## Abstract (web summary)

Changes in osmotic homeostasis alter metabolites and therefore chemical milieu of the cells. Here, the authors show that altering metabolites in *E. coli* also change the cellular capacity for buffering mutations that impair protein folding and influences proteostasis irrespective of molecular chaperones

## Introduction

Metabolic rewiring is a common response among different organisms to their surrounding environment^1^. Different cell types differ in their preferred mode of metabolism in order to harness energy and generate its required set of metabolites^2–5^. Interestingly, many of the cellular metabolites are known to modify protein stability and folding kinetics in vitro at high concentrations. Some of the special ones like polyphosphates^6,7^ and ATP^8,9^ are known to affect cellular protein aggregation during certain stresses. It is important to know if change in metabolite composition of the cell can alter intracellular protein folding capacity.

If metabolism can affect protein folding, it may have two fundamental implications. (1) Metabolism-dependent change in proteostasis may aid proteome evolution when there is a change in an organism’s niche or surrounding climate, or when an organism undergoes a large change in metabolism^4,10^. For example, some mutations may be rendered inactive in one metabolic state (a metabolic state is defined by the concentration of each metabolite the cell accumulates), while being active in a different one. Switching niches may expose certain phenotypes that are hidden by metabolism-dependent mutational buffering. (2) tissue-specific metabolic differences may predispose cell types to aggregate or misfold particular mutant protein, even while the protein is ubiquitously expressed. Age-dependent change in metabolism may also render certain tissues more prone to age-dependent aggregation^11,12^. This may have implications in explaining the late-onset tissue specificity of aggregation associated disorders^13^, which has been hard to explain with our current understanding of proteostasis components.

To test the fundamental link between metabolites and protein folding, we chose *E. coli* as our model organism, as it is well characterized in terms of its metabolic and protein quality control networks, and has simple mechanisms for chaperone induction^14,15^. One of the ways protein folding can be studied is by monitoring the capacity of the cells to buffer nonsynonymous mutations^16,17^. Although there is mixed evidence in the literature suggesting that chaperones assist mutational buffering^18–22^, little is known about the contribution of cellular metabolites for the same. Previous reports showed that the addition of small molecules at large concentrations in growth media leads to mutational buffering in a small-molecule dependent and mutant-specific manner shaping molecular evolution^17,23,24^. However, we do not understand if the physiological concentrations of metabolites present inside the cell can affect protein folding and mutational buffering.

Cells respond to osmotic shock by rewiring metabolism^10,25^ which allows them to accumulate compensatory osmolytes^26^. Osmolytes also influence protein stability in vitro^24,27–30^. We hypothesized that change in the osmotic composition of a cell may influence protein folding, and mutational buffering. To test this, we have used strains with altered levels of intracellular osmolytes and monitored their potential to buffer mutations in two model proteins. Indeed, the mutational buffering capacity differs with change in the metabolite pools. The buffering capacity of the same strain in different metabolic states is different. In all cases, mutational buffering is only evident for mutations that impair folding, corroborating the link between protein folding and genetic buffering. Remarkably, the metabolites that change along with buffering capacity can aid protein folding in vitro, suggesting a strong link between metabolite-assisted protein folding and genetic buffering. Finally, we demonstrate the link between metabolic state and mutational buffering by evolving strains of *E. coli* with enhanced osmotic tolerance. These strains show similar altered buffering capacity as seen for metabolically compromised cells, highlighting that the protein folding environment is different in different metabolic states. We propose that metabolic alterations can have far-reaching consequences on mutational buffering through their influence on cellular protein folding and proteostasis capacity.

## Results

### Altered metabolite uptake affects mutational buffering

**Fig. 1:**
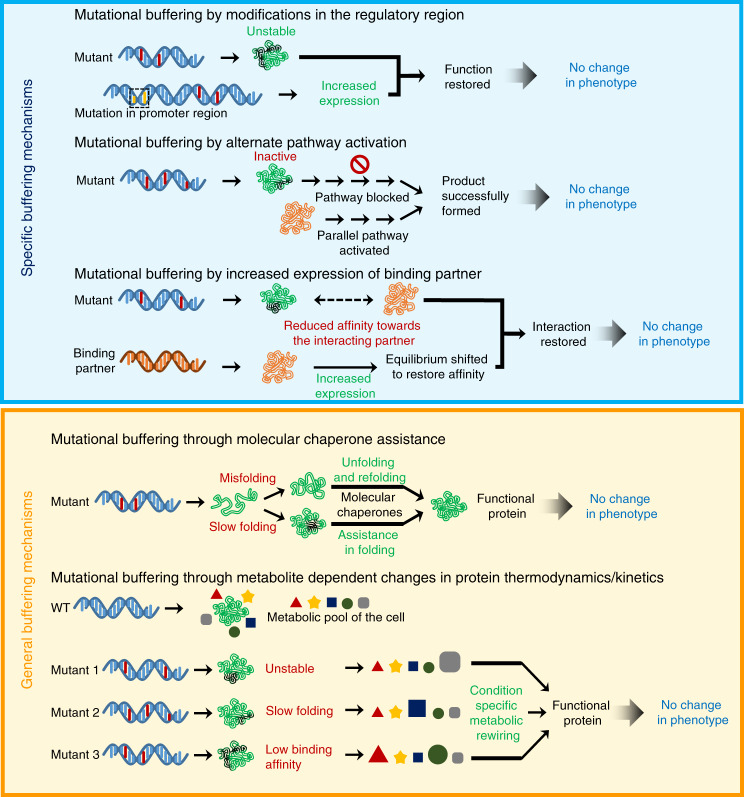
Schematic showing specific and general buffering mechanisms. Mutational buffering in a cell can be achieved by modifications in the regulatory region of a substrate leading to its increased expression, activation of alternate pathways or by increased expression of the binding partner for a specific substrate (top panel). However, generalized buffering of mutant phenotypes is executed by protein folding assistance through molecular chaperones or through relative composition and concentration of metabolite and resultant changes in protein thermodynamics and kinetics (bottom panel).

To elucidate if metabolic rewiring changes cellular capacity to buffer mutations, we used two model proteins- Gentamicin-acetyl transferase (Gm-R, confers gentamicin resistance)^31^ and Green Fluorescence Protein (GFP—yeast enhanced variant)^32^. These proteins met a few essential requirements. (1) Employing these model proteins, we could monitor the activity of multiple mutants simultaneously. (2) These proteins are non-endogenous to *E. coli* and their activity is largely independent of endogenous *E. coli* gene regulatory network except for the proteostasis network that takes care of its biogenesis and degradation. It ensured that altered buffering of different mutants of the proteins in different conditions is due to alteration in the general mutational buffering capacity of *E. coli* (Fig. 1). Using endogenous proteins instead would complicate the study as buffering would happen in both general and protein-specific manner (Fig. 1). This was overcome by the use of exogenous proteins. (3) The two chosen proteins have unique protein-folds, presumably with different folding requirements. This enabled us to not only exclude the fold-specific artifacts but also increased the ability to observe the breadth of buffering of folding-compromised mutants. (4) GFP was amenable to in vitro protein folding studies, allowing us to reconstitute the buffering activities in vitro thereby helping us to delineate the molecular mechanism of buffering.

**Fig. 2:**
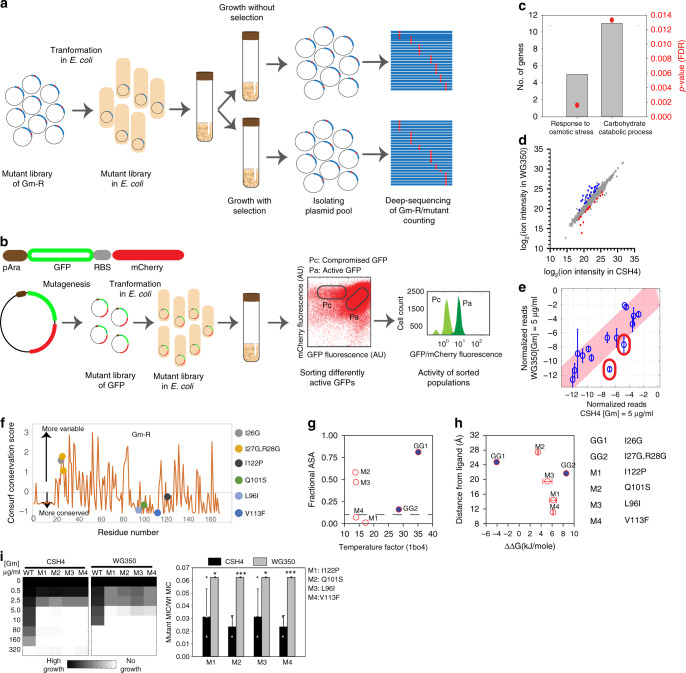
Genetic alteration in cellular metabolism changes mutational buffering. **a** Schematic for activity assay for glycine-doublet substitution library of Gm-R. Gm-R activity is inferred from competitive fitness of the mutants under gentamicin selection and deep-sequencing. **b** Schematic for activity assay of GFP-mutant library. An arabinose inducible promoter drives the bicistronic construct of GFP and mCherry. GFP is mutated using random mutagenesis. GFP mutants are sorted into population of compromised mutants (Pc) and active mutants (Pa) based on GFP Fluorescence. **c** Gene Ontology (GO) classes upregulated in WG350 transcriptome with respect to CSH4 shown as fold enrichment on the left-axis and Benjamini-Hochberg FDR corrected *p*-values obtained using DAVID^63^ on the right axis from two biological replicates. **d** Comparison of metabolites in WG350 and CSH4 using untargeted metabolomics. Colored circles represent significantly different metabolites between CSH4 and WG350 obtained using paired 2-sided Student’s *t*-test (*p*-value < 0.05, five biological replicates for each sample). **e** Mean of normalized read counts of Gm-R GG-mutant library at comparative selection pressure of gentamicin (Gm) from two biological replicates. Pink shaded area marks 99% confidence interval. Mutants marked in red show lower read counts in WG350 than in CSH4. **f** Conservation score (calculated using Consurf) for each residues that are mutated in the clones studied. **g** Fractional ASA (calculated using VADAR) of the mutated residues against the temperature factor of the residues obtained from PDB file 1BO4 (left panel). **h** Distance of the residues mutated from the ligand plotted against the predicted alteration in protein stability. Error bar represents standard deviation from three replicates (calculated using FoldX). **i** Heatmap representing growth of Gm-R mutants in WG350 and in CSH4 along with Wt Gm-R in increasing gentamicin (Gm) concentration (left panel). MIC of each mutant normalized with respect to Wt Gm-R in the respective strains (right panel). Mean is plotted with standard deviations as error bars from four biological replicates. Significance is calculated using 2-sided Students’ *t*-test with respect to CSH4 (*p-value M1:0.03, M3:0.03; ***p-value M2:0.0001, M4:0.0001). Also see Supplementary Fig. 1 (Source data are provided as source data file Fig. 2).

To generate a comprehensive map of mutational buffering, we developed massively-parallel activity assays to quantitate protein activity of a large number of mutants of the test proteins. Since Gm-R confers resistance to Gentamicin (Gm), we developed a high throughput activity assay for Gm-R based on deep-mutational scanning^16^. We used a Glycine doublet mutant (Gm-R GG) library for Gm-R^17^ (Fig. 2a, Supplementary Fig. 1A and 1B). For the second test protein GFP, where we could isolate clones with different levels of GFP fluorescence, we used a random mutant library of GFP. Quantification of the intracellular folded fraction of GFP was done using flow cytometry. Wt mCherry was expressed along with GFP as a bicistronic construct to control for differences in promoter activity and plasmid number (Fig. 2b).

To obtain *E. coli* strains with altered metabolism, we chose the strain CSH4^33^ and a mutant strain on this background deleted of Proline and Glycine-betaine uptake transporters (WG350) (Supplementary Table 1)^34^. We chose this mutant as it is osmosensitive as compared to CSH4 (Supplementary Fig. 1C) suggesting an altered concentration of intracellular osmolytes; this served as an experimental model for alteration in metabolism and osmolyte composition. To validate that the mutant strain WG350 had altered metabolism, we obtained mRNAs differentially expressed between WG350 and CSH4 using RNA-seq; which were significantly enriched for genes encoding metabolic enzymes (Fig. 2c). To substantiate if the strains differed in terms of metabolite concentrations, we compared their untargeted metabolite profiles (Fig. 2d, Supplementary Fig. 1D) and identified 43 metabolites showing differential abundance (*p*-value < 0.05). For example, Trehalose and Trehalose-6-Phosphate were increased by ~3 and ~4-fold, respectively, proline and betaine being marginally decreased (insignificant) in WG350 compared to CSH4 (Supplementary Table 2), indicating the cellular biosynthetic processes are switched on in the absence of transporters. For Glycine-betaine we found a coherent increase in the transcripts encoding genes for its endogenous synthesis and an increase in metabolites involved in its synthesis (Supplementary Fig. 1E). This demonstrated that cellular metabolism was rewired significantly upon the deletion of transporters for Proline and Glycine-betaine. Taken together, metabolism in CSH4 and WG350 strains differed significantly providing us the platform to ask if these strains also differed in mutational buffering.

To check for mutational buffering, we transformed both the strains with Gm-R GG library and grew them at similar selection pressure in the presence of Gentamicin (Supplementary Fig. 1F). We normalized for strain-specific differences in sensitivity to gentamicin by comparing activity of mutants with that of Wt Gm-R within the strain (Supplementary Fig. 1F). Mutant pools were sequenced and analyzed to obtain abundance as enrichment scores (measure of activity) of different mutants in presence and absence of Gentamicin^16^. Sequencing based enrichment scores correlated well with the minimum inhibitory concentration (MIC) of gentamicin-based semi-quantitative measurements of activity (Supplementary Fig. 1G). While most mutants were similarly active in the two stains CSH4 and WG350, two of the partially active Gm-R mutants (GG1 (I26G) and GG2 (I27G, R28G)) were less active in WG350 than in CSH4, indicating mutation-specific differences in buffering (Fig. 2e). We confirmed that transcription/translation was not different between these two strains using a bicistronic GFP/mCherry system (Supplementary Fig. 1H). The Gm-R mutations buffered differently between strains were in the non-conserved region of the protein (Fig. 2f), exposed to solvent, and flexible (Fig. 2g). One of the mutants was predicted to decrease the stability of the protein indicating folding problems; however, none of them were located near the active site (Fig. 2h). This suggested that alteration in metabolism may affect the folding of these mutants in WG350 strain. Remarkably, a different set of small-molecule-dependent Gm-R mutants isolated in a previous study^17^ show higher activity (w.r.t Wt Gm-R) in WG350 than in CSH4 (Fig. 2i, Supplementary Fig. 1b). All these mutant proteins harbored mutations in conserved residues (Fig. 2f), that were relatively inflexible (Fig. 2g). While these mutations were away from the ligand-binding site, all of them were predicted to partially destabilize the protein (Fig. 2h). This indicated that mutations even in the buried, conserved residues that destabilize protein structure could be buffered differently in the two strains with metabolic differences.

To compare mutational buffering between WG350 and CSH4 for the second test protein, we purified a population of GFP mutants (pool with compromised fluorescence, Pc) that showed lower GFP fluorescence compared to Wt GFP in WT *E. coli* strain BW25113 (referred to as BW henceforth). The mutant pool (Pc) had similar fluorescence in CSH4 and WG350 strains (Supplementary Fig. 1I), demonstrating that the difference between the strains in buffering mutations is protein-specific. Taken together, this suggested that the ability to take up metabolites from the medium affected metabolic network and mutational buffering. This changed the spectrum of mutations buffered in a protein-specific manner.

### Different metabolic states of the same cell show differences in buffering capacity

**Fig. 3:**
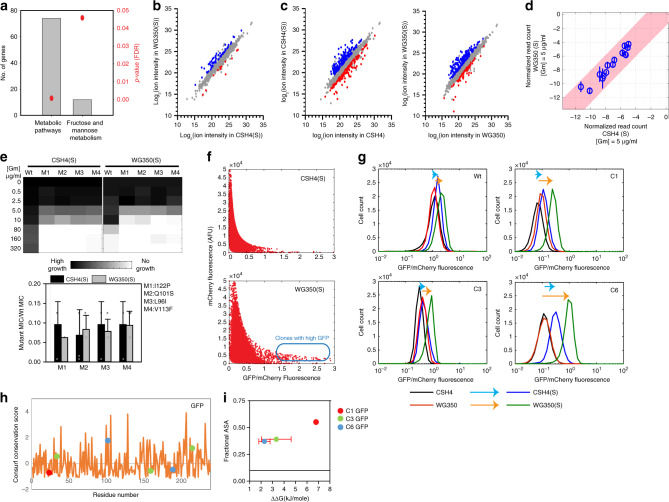
Osmotic stress-induced changes in metabolism changes the spectrum of mutants buffered. **a** Gene Ontology (GO) classes upregulated in WG350(S) transcriptome with respect to CSH4(S) shown as fold enrichment on the left-axis and Benjamini-Hochberg FDR corrected *p*-values obtained using DAVID on the right axis from two biological replicates. **b** Comparison of metabolites in WG350 and CSH4 during growth in media containing 350 mM NaCl using untargeted metabolomics. Colored circles represent significantly different metabolites obtained using paired 2-sided Student’s *t*-test. **c** Comparison of metabolites within strain in presence and absence of NaCl. Colored circles represent significantly different metabolites obtained using paired 2-sided Student’s *t*-test. **d** Mean of normalized read counts of Gm-R GG-mutant library at comparative selection pressure of Gentamicin (Gm) and 350 mM NaCl in growth media from two biological replicates. Pink shaded area marks 99% confidence interval. **e** Heatmap representing activity of small-molecule-dependent Gm-R mutants in CSH4(S) and WG350(S) at different concentrations of Gentamicin (Gm) (top panel). Bottom panel shows mutant MIC normalized with respect to Wt Gm-R in the respective strains. Mean with error bars representing standard deviation from four biological replicates is plotted (2-sided Student’s *t*-test *p-*value > 0.05). **f** The scatter plot for mCherry Vs GFP/mCherry fluorescence of pool of GFP mutants with compromised fluorescence (Pc) in WG350(S) and CSH4(S). Blue box indicates clones buffered in WG350(S). **g** Histogram of GFP/mCherry fluorescence of Wt, C1, C3, and C6 GFP in WG350 and CSH4 in presence and absence of osmotic stress. Cyan arrow indicates increase in GFP fluorescence upon addition of 350 mM NaCl to CSH4 strain, orange arrow indicates the same for WG350. **h** Conservation score (calculated using Consurf) for each residue. Dots indicate residues that are mutated in the clones studied. **i** Fractional ASA (calculated using VADAR) of the residues mutated in the clones is plotted against ΔΔG of the residues obtained from 1GFL. Error bars represent standard deviation from three replicates (calculated using FoldX). For **b** and **c**: *p*-value < 0.05, five biological replicates per sample. Also see Supplementary Fig. 2 (Source data are provided as source data file Fig. 3).

Next, we asked if altering the metabolite pool in the same strain background changes mutational buffering. Since osmotic shock facilitates the accumulation of osmotically active metabolites^25,26^, we grew the strains WG350 and CSH4 in 350 mM NaCl (CSH4(S) and WG350(S)). We obtained transcriptomic (Fig. 3a) and metabolomic shifts (Fig. 3b, Supplementary Fig. 2A) associated with osmotic shock in each of the strains. Osmotic shock altered metabolism in the two strains differently (Fig. 3c, Supplementary Fig. 2B). For example, levels of Fructose-1,6-bisphosphate increased in CSH4 upon osmotic shock but not in WG350, contrastingly, Succinate increased in WG350 but not in CSH4 upon osmotic stress (Supplementary Table 2).

Interestingly, the osmotic adaptation of CSH4 and WG350 strains led to a marked similarity in terms of their potential to buffer di-glycine mutations in Gm-R (Fig. 3d). GG1 (I26G) and GG2 (I27G, R28G), mutants less active in WG350 than in CSH4, showed similar activity in these strains in the presence of NaCl. The mutants of Gm-R that had enhanced activity in WG350 compared to CSH4, showed similar activity in both the strains during osmotic stress (Fig. 3e). This ruled out the canonical effect of osmotic stress-induced aggregation in affecting buffering under the conditions used here. The buffering capacity of WG350 and CSH4 towards Gm-R was similar although the osmotic composition of the strains in presence of salt was different. This indicated that compensatory mechanisms may work through different metabolic pathways to reinstate a similar spectrum of mutational buffering.

Since metabolite composition of CSH4 and WG350 differ in presence of NaCl, we asked if they also differ in buffering mutations on an unrelated protein, using GFP-mutant library (Fig. 3f). We identified multiple clones of GFP that showed enhanced fluorescence in WG350 compared to CSH4 in the presence of osmotic stress. This clearly showed that the activity of these GFP mutants was enhanced in the altered osmotic condition. In order to validate the observed buffering, we isolated single clones from the pool using FACS and sequenced them. The buffered pool isolated was enriched in mutation G24C (C1 GFP) amongst other mutations E34G, N159K, K214T (C3 GFP) and D102N, I188N (C6 GFP). (Supplementary Fig. 2C). Upon retransformation, each of these mutants exhibited similar fluorescence in CSH4 and WG350 in normal growth media while their fluorescence increased under osmotic stress (Fig. 3g, Supplementary Fig. 2D). Notably, these exhibited not only higher fluorescence in WG350 than in CSH4 in the presence of osmotic stress but also different mutants were buffered to different extent. Each of these clones had at least one mutation that mapped onto highly conserved region of the protein (Fig. 3h), was exposed and predicted to destabilize the protein structure (Fig. 3i). These clones confirmed that differences in metabolism could buffer mutations in conserved regions that tend to destabilize proteins, but in a protein-specific manner in different conditions.

### Mutational buffering is affected by protein folding capacity

**Fig. 4:**
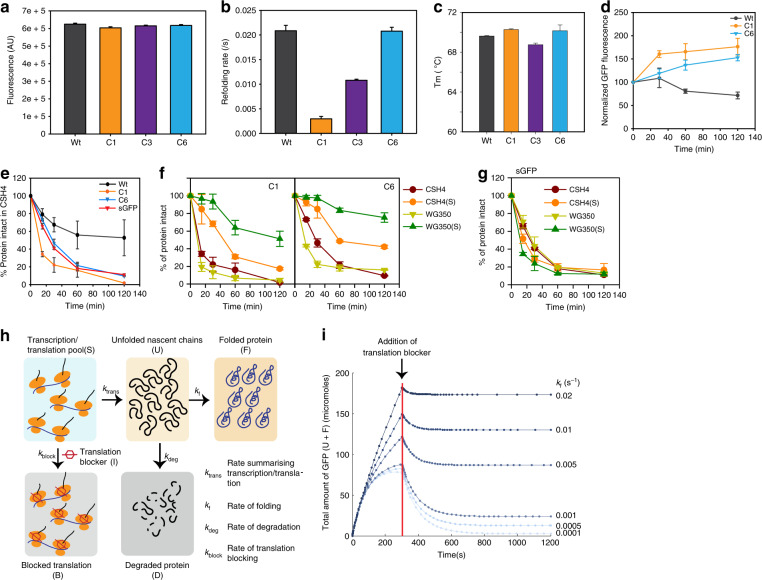
Mutations buffered by altered metabolic states have compromised protein folding. **a** Fluorescence of Wt, C1, C3, and C6 GFP at 515 nm and 200 nM concentration. Mean is plotted with standard deviation as error bars from four biological replicates. **b** Mean of spontaneous refolding rates of Wt and GFP mutants in Buffer-A obtained by fitting the refolding traces to single exponential equations. **c** Average (mean) melting temperature (Tm) calculated from thermal melts for Wt, C1, C3, and C6 GFP. **d** Mean of normalized GFP fluorescence of Wt, C1, and C6 GFP at 0, 30, 60, and 120 min post-translation arrest with Chloramphenicol after 5 h induction of GFP in CSH4. **e** Mean of gel-based quantification of intact GFP in Chloramphenicol-chase assay of Wt and GFP mutants in CSH4. **f** Mean of gel-based quantification of intact GFP in Chloramphenicol-chase assay of C1 and C6 GFP in CSH4, CSH4(S), WG350, and WG350(S). **g** Mean of gel-based quantification of intact GFP in Chloramphenicol-chase assay of sGFP in CSH4, CSH4(S), WG350, and WG350(S). **h** Schematic for numerical simulation with a fixed concentration of mRNA/ribosome complex (S). These complexes could either form nascent polypeptides (U) with rate k_trans_ (collective rate constant for transcription/translation), or inhibited with rate constant k_block_ in presence of a translation inhibitor (I). The pool U could either degrade with rate k_deg_ or fold with rate constant of k_f_ and reach the native state (F). Total amount of undegraded protein (U + F) after blocking translation was monitored after 300 s of starting the simulation (start of simulation mimics induction). **i** Total amount of intact GFP obtained from simulation as a function of k_f_ keeping k_deg_ constant at 0.01 s^−1^. Red line indicates the time-point at which 1 mM of translation inhibitor (I) is added. For **b**, **c**, **d**, **e**, **f**, and **g**: Standard deviations are plotted as error bars from 3 biological replicates. Also see Supplementary Fig. 3 (Source data are provided as source data file Fig. 4).

Having obtained different clones of GFP we asked if mutational buffering has contribution from altered proteostasis. The mutations did not map close to the fluorophore of GFP^35^ (Supplementary Fig. 3A) and in vitro fluorescence of the purified mutants was similar to Wt GFP (Fig. 4a) confirming that the mutations did not affect fluorescence of the folded proteins. Refolding studies of the purified proteins showed that the apparent rate for refolding was unchanged for C6 while C1 refolded with a rate ~10-fold slower and C3 refolded with a rate ~2-fold slower than Wt GFP (Fig. 4b, Supplementary Fig. 3B). However, the native state of C1 and C6 mutants were as stable as Wt GFP towards temperature denaturation (Fig. 4c, Supplementary Fig. 3C) while C3 was marginally destabilized, suggesting that defect in folding may depend on either the stability of the folding intermediates or that of the native state. This demonstrated that the mutants isolated were folding-compromised mutants resulting from energetic frustrations in the folding landscape. Thus, differences in their in vivo fluorescence may reflect the differences in protein folding capacities between strains.

To check for in vivo folding defects in the isolated GFP mutants, we looked at the degradation rates of a representative set of mutants (slow folding and buffered- C1; fast folding and buffered- C6) and Wt GFP by stalling translation (Supplementary Fig. 3D). First, we checked if the native state of the mutants were less stable than Wt GFP in vivo by monitoring the fluorescence of the mature protein after blocking translation (Fig. 4d, Supplementary Fig. 3E). Turnover of the mutants did not differ significantly from that of Wt GFP corroborating with the in vitro data that the native state of these proteins is similarly stable in vivo at physiological temperatures. Next, to check if folding intermediates of the mutants are less stable than Wt GFP we followed the turnover of nascent GFP polypeptides by blocking translation after briefly inducing protein expression (Fig. 4e). The total GFP level was monitored using immunoblotting. All the mutants degraded faster than Wt GFP underlining that nascent chains of these mutants were folding-compromised under in vivo conditions (Fig. 4e). This clearly demonstrated that the isolated mutants (C1 and C6) have folding intermediates that are less stable than the folding intermediates of Wt GFP resulting in compromised folding pathways in vivo.

**Fig. 5:**
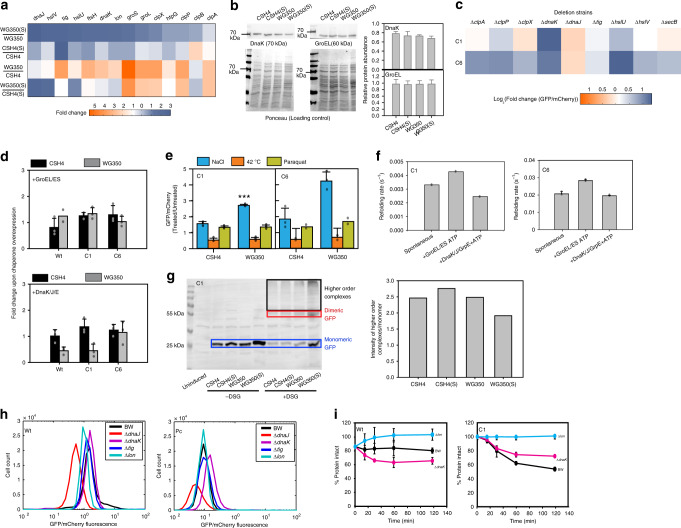
Folding of isolated mutants is independent of molecular chaperones. **a** Heatmap representing relative quantification of transcripts encoding proteostasis components in WG350 and CSH4 in presence and absence of 350 mM NaCl. **b** Immunoblot (left panel) and image-based quantification (right panel) of DnaK and GroEL in CSH4 and WG350 in presence and absence of 350 mM NaCl (*p*-value > 0.05). Mean is plotted with standard deviation as error bars from four biological replicates. **c** Heatmap representing Wt GFP normalized log_2_ fold-change in GFP/mCherry fluorescence in deletion strains against that in BW for Wt, C1, and C6 GFP. **d** Bar graph representing mean of GFP/mCherry fluorescence of Wt, C1, and C6 GFP in CSH4 or WG350 with and without plasmids overexpressing either GroEL/ES (top) or DnaK/J/E (bottom) (*p*-value > 0.05). **e** Bar graph representing GFP/mCherry fluorescence of Wt, C1, and C6 GFP in CSH4 and WG350 in absence or presence of external stressors (42 °C—heat shock, 80 μM Paraquat- oxidative stress, 350 mM NaCl - osmotic stress). Mean is plotted with significance calculated using 2-sided Students’ *t*-test (*p-value 0.017; ***p-value 0.00007). **f** Bar graph for mean of refolding rate for unfolded C1 and C6 GFP in presence and absence of 400 nM DnaK/800 nM DnaJ/400 nM GrpE or 400 nM GroEL/800 nM GroES. **g** Immunoblot for GFP showing recruitment of nascently formed C1 GFP into monomeric GFP (blue box), dimeric GFP (red box) and higher order complexes (black box). The ratio of GFP in higher order complex to the amount in monomeric state is shown in bar graph. **h** Histogram for GFP/mCherry fluorescence of Wt and pool of GFP mutants (Pc) in BW, molecular chaperone (∆*dnaJ,* ∆*dnaK,* ∆*tig*) and protease (∆*lon*) knockout strains. **i** Plot for quantification of intact GFP in Chloramphenicol-chase for Wt and C1 GFP in BW, ∆*dnaK*, and ∆*lon* strains. Mean from three biological replicates is plotted. For **d**, **e**, **f**, and **i**: Standard deviations are plotted as error bars from three biological replicates. Also see Supplementary Fig. 4 (Source data are provided as source data file Fig. 5).

The in vivo degradation rates of the mutant GFPs in CSH4 and WG350 were similar; however, there was a sharp decrease in degradation rates of the C1 and C6 in WG350(S) compared to CSH4(S) (Fig. 4f (graph), Supplementary Fig. 3F (gel)). This was not due to a general decrease in degradation capacity of the cell, as sGFP—a degradation prone mutant of GFP—degraded with similar kinetics in both the stains regardless of salt stress (Fig. 4g (graph), Supplementary Fig. 3H (gel)). Furthermore, the general proteases were upregulated in WG350(S) (Fig. 5a) arguing against a possible decrease in degradation capacity of WG350(S).

A simple kinetic simulation of protein synthesis followed by modeling competition between folding and quality control assisted degradation (Fig. 4h, Supplementary Table 3) indicated that an increase in folding rate would be expected to show a decrease in degradation (Fig. 4i). Thus, the apparent decrease in degradation rate may arise solely due to differences in the folding rate in vivo in different strains and conditions while the degradation capacity remains constant (as seen for sGFP). Taken together this indicated that the mutants isolated were indeed folding mutants and mutational buffering in the sensor proteins differed between the strains due to their differences in protein folding capacities.

### The mutants are dependent on chemical chaperones

To investigate the contribution of molecular chaperones, we checked if their levels changed in WG350(S). Interestingly, mRNA levels of most of the genes related to protein quality control (except DnaJ and HslU/V) (Fig. 5a) and protein levels of canonical chaperones GroEL and DnaK (Fig. 5b) were unchanged in WG350(S). Isolated GFP mutants did not show reduced in vivo fluorescence in absence of abundant canonical molecular chaperones (tig, dnaK, dnaJ, secB)^15^ or proteases (hslU/V, clpX/P/A)^36^, suggesting that folding of these mutants was independent of canonical proteostasis machinery (Fig. 5c). It would also mean that overexpression of DnaJ or HslU/V seen in WG350(S) at mRNA levels may not contribute to the mutational buffering of the mutants. Coherently, overexpressing GroEL/GroES or the DnaK/DnaJ/GrpE in CSH4 or WG350 did not increase the fluorescence of these mutants (Fig. 5d, Supplementary Fig. 4A, 4B), suggesting that the buffering effect in WG350(S) was independent of the concentration of these molecular chaperones. To mimic a global increase in chaperone levels, we used other environmental stressors like heat shock and oxidative stress that are known to increase stress-response driven chaperone levels^15,37,38^. The fluorescence of GFP mutants was only enhanced with osmotic stress and not with heat or oxidative stress (Fig. 5e).

In vitro, GroEL/ES machinery could only marginally accelerate refolding of GFP mutants C1 and C6, whereas DnaK/DnaJ/GrpE did not (Fig. 5f, Supplementary Fig. 4C). This confirmed that these mutants are not substrates of the abundant chaperone machinery in vivo and in vitro. To investigate potential interactions of GFP mutants with any other molecular chaperones, we performed in vivo crosslinking to probe higher order complexes formed by GFP mutants in CSH4 and WG350, with and without osmotic stress (Fig. 5g, Supplementary Fig. 4d). The levels of higher order complexes were not increased in WG350(S), indicating that the chaperone association was not altered. Thus, strain-specific protein folding differences that alter mutational buffering had contributions from components other than molecular chaperones.

To demonstrate that the mutants buffered in different metabolic states were indeed special in terms of their independence of chaperones and not an artifact of chaperone independence of the model proteins chosen, we checked the chaperone dependence of the partially compromised (Pc) population of GFP library in molecular chaperone deletion strains (Fig. 5h, Supplementary Fig. 4e). The mutant pool (Pc), as well as Wt GFP, showed decreased fluorescence indicating that Wt GFP itself is recognized by DnaJ. Fluorescence of the mutant pool increased in Δ*dnaK*. This suggested that DnaK could recognize and hold the mutant proteins and prevent their folding. Additionally, we were able to isolate a GroEL/ES-dependent mutant of GFP from this pool^39^, proving that the GFP-mutant pool consisted of mutants efficiently recognized by the endogenous chaperone systems. Thus, the mutant library had mutants that were dependent on the molecular chaperones although these were not differently active in the different metabolic states of *E. coli* we tested. Next, we checked if the isolated mutants C1 and C6 were efficiently recognized by the most abundant chaperone system. Chloramphenicol-chase experiment with C1 and C6 in BW, Δ*dnaK*, and Δ*lon* strains (Fig. 5i, Supplementary Fig. 4F) clearly showed that the mutant proteins are recognized by DnaK chaperone system and effectively degraded only in presence of DnaK and Lon. Taken together this showed that the mutant library of GFP, as well as the mutants identified to be differently buffered in different metabolic states, were recognized by the endogenous chaperone system. We also compared the activity of the complete Gm-R GG library as well as two mutants in Δ*dnaK* strain with that in BW (Supplementary Fig. 4G). The pool, as well as two isolated mutants, showed decreased Gm-R activity in absence of DnaK indicating that DnaK assists in the folding of some of the Gm-R mutants. Thus, both the mutant pools (GFP and Gm-R) tested for mutational buffering contained mutants that were dependent on the endogenous chaperones but the difference in activity of the selected mutants in different metabolic states was not due to difference in concentrations of molecular chaperones between the two strains.

**Fig. 6:**
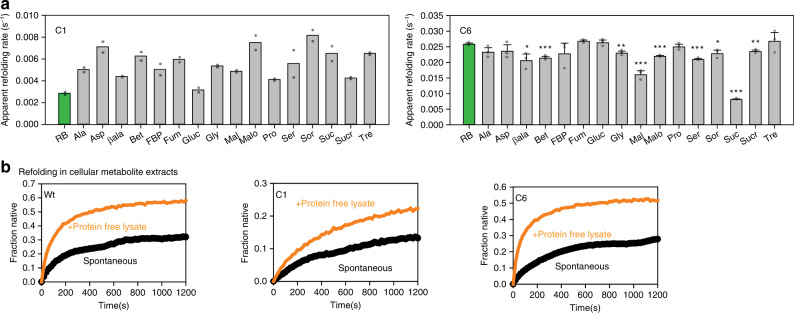
Metabolites can chaperone the mutants in vivo and in vitro. **a** Refolding of Wt GFP or mutant GFP was initiated as described earlier in the presence of 100 mM concentration of different metabolites. Refolding rates were obtained by fitting the refolding traces with single exponential equations. Mean of refolding rates from two replicates for C1 and three replicates for C6 along with standard deviation as error bars. RB (shown in green bars) are refolding rates in Buffer-A, the base buffer in which the metabolites are added. *p*-value is calculated using 2-sided Students’ *t*-test with respect to RB (*p-value βala :0.02, Sor: 0.02; **p-value Gly: 0.006, Sucr: 0.005; ***p-value Mal: 0.0004, Bet: 0.0007, Malo:0.0002, Ser: 0.0001, Suc:0.0000005). Ala Alanine, Asp Aspartate, βala β alanine, Bet Betaine, FBP Fructose-1,6-bisphosphate, Fum Fumarate, Gluc Glucose, Gly Glycine, Mal Malate, Malo Malonate, Pro Proline, Ser Serine, Sor Sorbitol, Suc Succinate, Sucr Sucrose, Tre Trehalose. **b** Protein-free extracts were isolated from wild-type *E. coli* (BW25113, BW) as described in Methods. Refolding was performed by diluting unfolded proteins (Wt GFP or mutants) 100-fold into these extracts. Also see Supplementary Fig. 5 (Source data are provided as source data file Fig. 6).

Mutant-specificity and protein-specificity of folding assistance in vivo are a hallmark of chemical-chaperone mediated folding. To confirm if these mutants were dependent upon chemical chaperones for folding, we obtained their refolding rates in presence of different metabolites (Fig. 6a). Many of the metabolites could act as chemical chaperones to accelerate refolding of C1 GFP like Aspartate and Glycine; C6 mutant did not show an enhanced refolding rate with any of the small molecules tested. This reiterated the mutant-specific effect of different chemical chaperones^17,24^ and suggested that these small molecules could facilitate the folding of these proteins and contribute to mutational buffering. Of note, the space sampled in terms of cellular small-molecules was non-exhaustive and no combinations were tried. Importantly, some of the small molecules (like Aspartate) were also able to increase the thermal stability of the mutants specifically (Supplementary Fig. 5A) indicating that these molecules could act in a mutant-specific manner to change energetics of folding. As a control, NaCl or KCl did not have any significant effect on C6 or C3, showing that salt used for osmotic stress did not affect the thermodynamics of folding for buffered mutants in general.

To check if small-molecule milieu of cells can aid folding, we reconstituted in vitro refolding of the selected GFP mutants in small-molecule enriched cellular extract of WG350. Refolding of GuHCl-unfolded GFP mutants was initiated by diluting out the denaturant in presence of extract obtained from WG350 (Fig. 6b). Interestingly, these mutants were refolded to only a negligible extent in absence of extract while folding substantially in its presence, underlining the importance of small-molecule milieu of the cell in chaperoning protein folding. We confirmed that these lysates were free from proteins and hence molecular chaperones that are known to assist refolding. It is important to stress that the current technologies of extraction limit concentration at which these small molecules can be extracted; they are more than a thousand-fold diluted from their physiological concentrations. It is therefore difficult to recapitulate the full potential of this mixture and reproduce the in vivo differences between strains. However, it demonstrated that the metabolite pool, even on dilution, acts as chaperone and hence could participate in cellular protein folding. Therefore, metabolic differences have the capacity to show altered mutational buffering in different metabolic states. This was primarily evident for mutations that destabilize the sensor proteins and make them sensitive to osmolyte concentrations. Taken together, metabolic differences manifested differences in mutational buffering, which may have a significant contribution from metabolite-dependent protein folding.

### Altering cellular metabolism changes mutational buffering

**Fig. 7:**
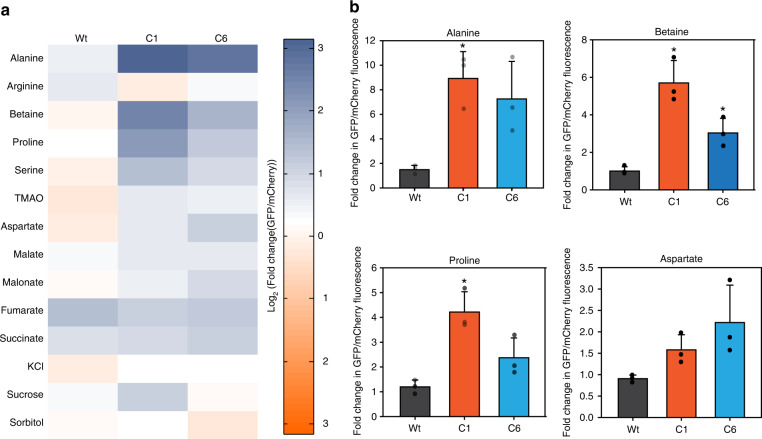
Altering metabolism by exogenous supplementation modifies mutational buffering capacity. **a** Expression of Wt, C1, and C6 GFP mutants was induced in CSH4 cells either growing in LB or in LB containing different metabolites. Average of log2 of fold-change in median of GFP/mCherry fluorescence as measured by single-cell fluorescence from three replicates is shown as heatmap. **b** Mean of fold-change in median of GFP/mCherry fluorescence in few metabolites (Alanine, Betaine, Proline, Aspartate) in CSH4 is shown as bar graph along with standard deviations from three biological replicates and *P*-value calculated with respect to Wt GFP by 2-sided Students’ *t*-test, *p-value C1 Alanine: 0.02, Betaine: 0.01, Proline: 0.01; C6 Betaine: 0.03. (Source data are provided as source data file Fig. 7).

Having realized that genetic and osmotic alteration in metabolism changes the spectrum of mutational buffering, we checked for altered buffering through metabolic changes obtained by adding excess of amino acids, sugars, and other metabolites to rich media, known to rewire metabolism^40,41^. Exogenous addition of many of these compounds to growth media (concentrations provided in Supplementary Table 4) led to enhanced folding of GFP mutants in CSH4 in vivo (Fig. 7a). Specifically, addition of Alanine increased the fluorescence of both the mutant proteins as seen in vitro (Fig. 7b). Further demonstrating that the mutants isolated from the screen predominantly responded to alterations in cellular protein folding capacity due to differences in metabolites, and mutational buffering could be altered by altering cellular concentration of metabolites. Interestingly, different additives had mutant-specific chaperoning activity in vivo as seen in vitro. This unraveled the complex connection between mutational buffering and metabolic status.

Taken together with our previous observation that these mutants are primarily dependent upon chemical chaperones for folding, we posit a prominent role of metabolism and metabolites in regulating differences in protein folding capacity.

### Osmotic composition determines mutational buffering

**Fig. 8:**
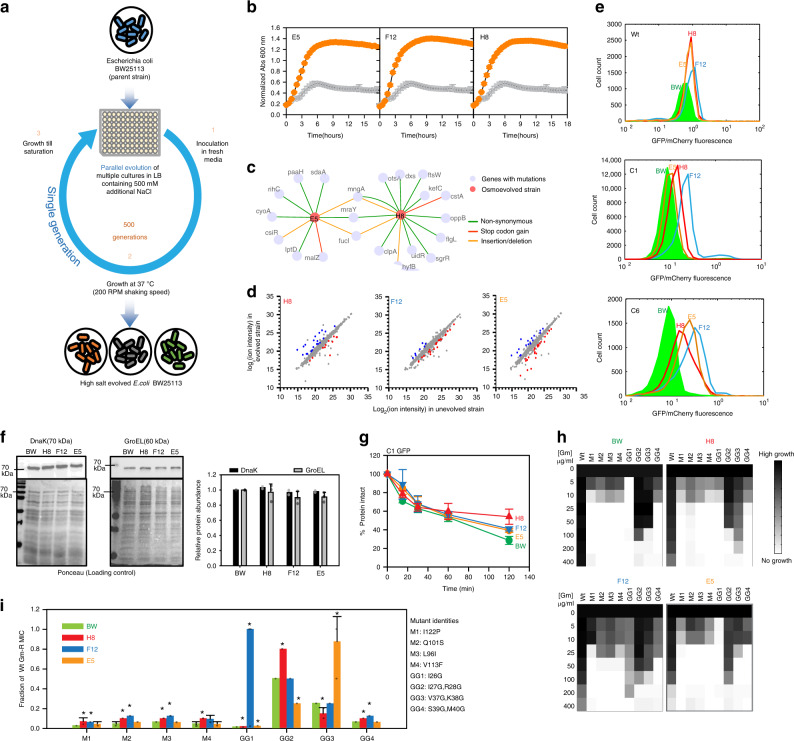
Wild-type cells evolve altered buffering capacity with evolution of a different metabolome. **a** Schematic of strategy for Laboratory Adaptive Evolution of osmotolerant strains of *E. coli* starting from BW (wild-type E. coli K-12, BW25113). **b** Growth curve of unevolved BW and evolved osmotolerant strains E5, F12, and H8 in 500 mM of NaCl added in excess to LB medium while growing at 37 °C, 200 rpm. **c** Genetic interaction network map of evolved strains E5 and H8 based on mutations obtained in genome sequencing. **d** Metabolite features of representative evolved osmotolerant strains (E5, F12, and H8) and its comparison with BW. The colored circled represent metabolites that are significantly altered in the evolved strains. **e** Histogram for GFP/mCherry fluorescence of Wt, C1, and C6 GFP in the BW and evolved strains (E5, F12, and H8). **f** Comparative quantification of chaperone proteins DnaK and GroEL in BW and evolved strains (E5, F12, and H8) done by immunoblotting with specific antibodies. Quantification of the chaperone levels are shown as bar graphs representing mean values (2-sided Student’s *t*-test *p*-value > 0.05). **g**. Chloramphenicol based chase for checking protein degradation rates were performed in BW and evolved strains (E5, F12, and H8) as discussed earlier. Mean of C1 GFP levels at different time points are shown in different strains. **h** Heatmap for growth-based activity of Gm-R mutants and Wt Gm-R in unevolved strain (BW) and evolved strains (E5, F12, and H8) in increasing concentrations of gentamicin. **i** Bar graph representing percentage MIC of a selected set of Gm-R mutants w.r.t Wt Gm-R in the respective strains. Mean of four biological replicates is plotted with standard deviation as error bars (unpaired 2-sided Student’s *t*-test *p-value for M1(H8:0.04, F12:0); M2(H8:0.002, F12:0.0002); M3(H8, F12:0); M4(H8:0.002); GG1(F8, F12, E5:0); GG2(H8, E5:0); GG3(H8:0.01, E5:0.002); GG4(H8, F12:0). For **b**, **f**, and **g**: Standard deviations are plotted as error bars from 3 biological replicates. Also see Supplementary Fig. 6 (Source data are provided as source data file Fig. 8).

Is the link between metabolism and mutational buffering relevant in the context of natural evolution? To answer this, we evolved BW strain to tolerate high salt stress and checked if this can be associated to an altered buffering capacity (Fig. 8a). The strain was continuously passaged in presence of 500 mM of NaCl added to LB. We kept the evolution duration short to ensure minimal drift mutations. Multiple strains were generated by parallel adaptive laboratory evolution and by ~500 generations, we obtained strains that were fitter in the presence of osmotic stress (Fig. 8b). We argued that adaptation for growth in chronic hyperosmotic stress would have fixed mutations that perturb the osmotic composition and hence rewired the metabolic status of the cell. Genome sequencing of two of these strains demonstrated accumulation of multiple different mutations (Fig. 8c, Supplementary Table 5) including genes involved in osmolyte synthesis (sdaA (gluconeogenesis), fucI (fructose and mannose metabolism), otsA (trehalose metabolism)). This indicated that the strains may have fixed certain traits associated with osmotolerance by acquiring mutations. We confirmed that these strains differ in metabolite pool even when grown in the absence of osmotic stress (Fig. 8d) and asked if these metabolic differences changed their mutational buffering capacity. Remarkably, the strains tested showed buffering for the C1 and C6 mutants of GFP that were buffered in WG350(S) (Fig. 8e). Activities of the mutants were two to four-fold higher in different evolved strains in a mutant-specific manner though folding of Wt GFP did not change in these strains (Fig. 8e). Moreover, *E. coli* molecular chaperones DnaK and GroEL were not upregulated in the evolved strains compared to the unevolved BW strain as checked by immunoblotting (Fig. 8f) indicating that canonical hubs of proteostasis were not altered in these evolved strains. The degradation of C1 GFP was slower in the evolved strains than in the unevolved strain (Fig. 8g, Supplementary Fig. 6B) independent of the activity of degradation machinery as confirmed by sGFP (Supplementary Fig. 6C); impaired degradation hence is a fallout of faster folding of the buffered GFP mutants in the evolved strains. This indicated that a genetically different strain evolved to have altered osmotic composition is able to buffer similar mutational variation as seen in the WG350 strain during osmotic stress. Response to osmotic stress once fixed in the genome, could buffer similar mutational variations even in absence of the stress. Like GFP mutants, mutants of Gm-R also exhibited higher activity in most of the evolved strains than unevolved BW (Fig. 8h, i). Importantly, each of the mutants had different activities in different osmotolerant strains. Taken together the metabolic state of a cell was directly linked to their ability to buffer mutations. Adaptation to a niche with different osmolarity changes the spectrum of mutations buffered. This, in turn, could change the route of molecular evolution of proteins, linking metabolism to the evolution of protein sequences through alterations in protein folding capacity.

## Discussion

In this work, we have shown the capability of *E. coli*, in affecting mutational buffering through accumulation of small molecules in the cell. We show this using two different proteins. However, there may be more complicated dependencies between different proteins and small molecules that affect their folding, that need to be unraveled. This work specifically describes chaperoning activity of the small-molecule component of the cytosol of *E. coli*. While this fact is important, its regulation through osmotic stress or genetic alterations makes it even more interesting in the context of regulation of protein folding, enabling condition specific canalization or decanalization of mutants^42–45^. It is known that perturbing growth conditions by exogenous supplementation by osmolytes can alter mutational buffering, specifically for mutations that affect either protein stability orfunctions^16^. Similarly, endogenous alteration in production of metabolites may also change mutational buffering. This we believe would be important when organisms shift niches and evolve new functionalities. It is tempting to speculate that conditions reported to alter Hsp90 levels during the evolution of *A. mexicanus* cave dwelling variants, may also have led to alteration in intracellular osmolyte composition^46^. While *E. coli* is a primitive model organism to comment on adaptive strategies and genetic buffering in higher eukaryotes with complex developmental pathways, it surely paves way for more interesting investigations to establish the potential link between osmotic composition, and their alterations with canalization.

Given that many of the small molecules that accumulate in cells like Arginine^24^, glycerol^47–49^, amino acids^24,50,51^ are reported to change protein folding kinetics and thermodynamics in vitro, it may be the complex milieu of metabolites and not osmolytes alone that can alter the folding process in a protein-specific manner. Although worked on for decades, we are yet to understand the molecular basis of protein stabilization by different osmolytes and metabolites. Their mechanisms seem to depend on the test protein, as well as on the specific type of problems that mutants encounter while folding^24^. While many of these molecules (like amino acids and sugars)^52,53^ can stabilize certain proteins by decreasing flexibility of non-native states, others may prevent formation of non-native contacts or intermolecular protein aggregation by partially destabilizing folding intermediates. Their contribution to cellular protein folding would require further investigation given our observations.

Alteration of metabolic states, as shown in this work provides another avenue for alteration of proteostasis. Using *E. coli* as test tube in our case we tested that composition of cellular milieu alters with metabolism, and has the potential to alter protein folding and proteostasis network. This links metabolism to protein folding in prokaryotes and implies that microbial evolution may be shaped differently when metabolism is altered either due to environmental pressure or due to genetic changes. Further work will be required to understand if similar mechanisms are prevalent in higher organisms.

## Methods

### Strains, plasmids, and proteins

*E. coli* strain DH5α was used for cloning, WT *E. coli* (K-12 BW25113 referred to as BW) strain was used for expression of arabinose inducible pBAD GFP mCherry and BL21 (DE3) was used for protein expression and purification. Protein concentrations were determined spectrophometrically at 562 nm using BCA kit (Pierce ThermoFisher Scientific). Deletion strains were obtained from CGSC as part of Keio collection^33,34,36^.

### Construction of mutant GFP-mutant library

Mutant GFP library was made in arabinose inducible pBAD vector using random mutagenesis approach. In the first step, yeGFP (referred to as GFP) was amplified using GFP specific primers and Mutazyme II polymerase (Agilent Technologies; Cat no. 200550) in order to incorporate 7–11 mutations per kb of plasmid. In the second step, the product of first amplification was used as mega-primer to amplify the entire plasmid with Wt GFP (pBAD GFP mCherry) as template using Kapa Biosystems Hifi readymix (NC02955239) for 25 cycles with both annealing and extension at 72 °C. The Wt copy of plasmid was digested with DpnI followed by transformation of chemically competent DH5α cells. The colonies were scraped and plasmid was prepared in pool to yield library of GFP mutants. The said library has a total complexity of around 10,000 mutations. The reporter is constructed such that GFP and mCherry are under same arabinose inducible pBAD promoter in an operon to give readout of GFP according to the mutation created on it but the mCherry readout will remain similar thus serving as an internal control for transcription, translation, and inducibility.

### Screening of mutant GFP library for folding mutants responsive to osmotic stress

WT *E. coli* cells (BW) were transformed with mutant GFP library maintaining 10-fold converge for preserving mutant library complexity. Cells were induced with 0.1% arabinose at the time of inoculation and fluorescence was observed on BD LSR II 5 h post induction at 37 °C after diluting cells in 1X PBS and incubating at 37 °C for 1 h. Fluorescence of the mutant library was studied in a pooled manner against Wt GFP. The entire library was sorted using BD Aria III into populations of compromised mutants (low fluorescent) and active mutants (high fluorescent) according to the GFP fluorescence. Each of these populations was purified and plasmids prepared. CSH4 and WG350 strains were transformed with the purified populations and subjected to osmotic stress with 350 mM NaCl. The pool of mutants buffered under osmotic stress in WG350 was sorted and single clones of GFP were picked from here and checked for their fluorescence in presence and absence of osmotic stress. The isolated mutants having higher fluorescence in WG350 under osmotic stress were identified by Sanger’s sequencing, and cloned in pET SUMO under BamHI and HindIII restriction sites and protein expressed in *E. coli* BL21 (DE3) were purified for further characterization.

### Growth curve

Single colony of *E. coli* cells were inoculated in LB and grown overnight at 37 °C, 200 rpm. Secondary inoculations were done in LB (control) and LB containing 350 mM, 500 mM NaCl additional in honey comb plates with temperature maintained at 37 °C, 200 rpm shaking. Absorbance at 600 nm was measured every 30 min using Bioscreen C (Oy growth curve Ab Ltd).

### Transcriptomics

Three milliliter of culture grown in LB overnight at 37 °C from a single colony was used to re-inoculate at 0.1% culture in 10 ml of LB and grown till OD600 reaches 0.5. The culture was mixed with equal volume of bacteria RNA protect reagent followed by RNA isolation using Qiagen RNeasy Mini Kit and TURBO DNA-free kit (AM1907). Quality of RNA was checked using RNA 600 Nano bioanalyzer kit and MICROB Express bacterial mRNA enrichment kit (AM1905) was used to remove rRNA. Hundred nanogram of RNA was used to prepare library using Ion Total RNA-seq kit V2 (4475936) and Ion Xpress RNA-seq barcode 1–16 kit. Final quality check and quantification was done using DNA HS bioanalyzer (5067-4626) and qubit HS DNA kit (Q32854). Equal amount of each sample was pooled followed by emulsion PCR (Ion PI Hi-Q OT2 200) and sequencing (Ion PI Hi-Q sequencing 200 kit) FastQC-tool kit was used for Data QC followed by Trimmomatic^54^ to remove low quality reads. TPM was calculated using Kallisto^55,56^ followed by identification of differentially expressed genes using EB sequencing analysis pipeline (https://bioconductor.org/packages/release/bioc/html/EBSeq.html)^57^.

### Metabolomics

Three milliliter of LB was inoculated with 0.1% inoculum from overnight grown culture and grown till OD_600_ 0.8 at 37 °C, 200 rpm. Cells equivalent to 0.1 OD were harvested at 17,530 × *g*, 1 min, 4 °C. Supernatant was discarded and 200ul chilled extraction solvent (80% MetOH in MS grade water containing1ng/µl PIPES and U13C-U15N-glutamine as internal standard) was added to the pellet, mixed and incubated in ice for 5 min for quenching metabolism. This is followed by sonication in water bath for 15 min at 4 °C with intermittent vortexing. Metabolites were collected in the supernatant by centrifugation at 17,530 × *g* for 5 min at 4 °C. Previous step was repeated twice by adding 100 µl 80% chilled solvent each time to increase metabolite yield and pooled extracts were stored in −80 °C refrigerator till further analysis. The untargeted mass profile (or metabolic profile) was acquired in Thermo Q-exactive Orbitrap coupled with Thermo Accucore RP C18 150*2.1, 2.6 μM column with flow rate of 200 μl/min. Ion masses from 80–1000 *m/z* was collected in negative ion mode. The raw output files obtained from mass spec were converted into.mzXML files using proteowizard tool (http://proteowizard.sourceforge.net/) followed by identification and analysis using metabolomics data processing software platform XCMS and MAVEN(http://genomics-pubs.princeton.edu/mzroll/index.php?show=index)^58,59^.

### Minimum inhibitory concentration (MIC) study of Gm-R glycine-doublet mutants

The library of Gm-R glycine-doublet mutants (Bandyopadhyay et al., 2012) were grown overnight in LB containing ampicillin (100 μg/ml) and Arabinose (0.1%) at 37 °C, 200 rpm. Secondary inoculations were done in LB containing Ampicillin (100 μg/ml), Arabinose (0.1%) and increasing concentrations of gentamicin (0–800 μg/ml) and incubated for 16 h at 37 °C and 200 rpm. Growth was assessed by measuring absorbance at 600 nm in flat bottom 96 well microtiter plate using TECAN infinite 200 pro. Absorbance value for each sample under selection pressure by gentamicin was normalized against absorbance of respective unselected sample. To obtain a semi-quantitative indication of the activity of different mutants, Wt Gm-R transformed cells were grown as control, and the MIC for the mutants was normalized with respect to the MIC of Wt Gm-R in the same strain.

### Amplicon sequencing

Plasmids were isolated from unselected (no gentamicin) and selected cells under gentamicin selection pressure. Gm-R gene was amplified for 25 cycles using Kapa Hifi Hotstart polymerase. The 500 bp amplicons were gel purified and quantified using qubit. One hundred and fifty nanogram of purified products was used for library preparation Ion Plus Fragment Library Kit (part no.4471252) and Ion Xpress Barcode Adapters (4471250, 4474009). The final library was quantified and equal amount of DNA library of eight samples were pooled and emulsion PCR and sequenced using Ion PGM Hi-Q OT2 Kit (part no A29900) and Ion PGM Hi-Q sequencing kit (part no. A30044) on Ion Torrent platform. Analysis was done using FastqQC followed by trimmomatic (cutoff Q15) and quality check by fastQC. Good reads were aligned to Gm-R gene followed by variant calling and fitness score calculation www.github.com/kc-lab/dms2dfe^16^.

### Quantitation of activity of Gm-R glycine-doublet substitution mutants

To quantitate activity of the mutants, we obtained the read count for each of the mutant and normalized it with respect to the coverage at the respective positions. The relative read counts of the mutants did not differ between the different strains in the absence of any selection pressure for Gm-R indicating that the library was homogeneously covered in all the transformations. We thereby did not obtain relative enrichment but compared the coverage-normalized read-counts between the different strains and conditions to obtain differences in activity. All the amplicon-based sequencing experiments were done in duplicates.

To obtain relative activity of the different mutants in one strain (for comparison of activity of mutants as measured by amplicon sequencing and MIC assay as shown in Fig. S1F) we obtained the enrichment score for each mutant by dividing the normalized read count for each mutant in presence and absence of gentamicin selection. z-scores were obtained for each of the mutant assuming a normal distribution. A positive z-score indicates higher than average activity while a negative z-score indicated a lower than average activity. Mutants were picked that had a high z-score (1.4, more active than average) or low z-score (−1.6 less active than average) or one close to zero (z-score = −0.4, activity close to average) for checking the MICs. Note: z-score was not used to obtain the significance of difference but the mutants that exhibited either high or low activity.

### Chase for degradation of protein

From overnight grown culture in LB containing Ampicillin (100 μg/ml) secondary cultures were inoculated with 0.1% inoculum in 100 ml LB containing Ampicillin (100 μg/ml) and grown till OD_600_ 0.5 or for 5 h (with inducer for chase of folded protein as shown in Fig. S3E). Cells were harvested and resuspended in 10 ml of the spent media (or media containing 350 mM NaCl added in excess to LB. GFP was induced using 0.5% arabinose for 5 min at 37 °C and 200 rpm. Chloramphenicol (50 μg/ml) was used to arrest translation. One milliliter of culture was taken out for uninduced, 0, 15, 30, 60, and 120 min post-translation arrest, harvested, snap frozen, and protein was collected by lysing cells resuspended in 1X PBS using 2X lysis buffer (Tris pH 6.8 (80 mM), SDS 1%, glycerol 10%). Thirty microgram of protein estimated using Pierce™ BCA Protein Assay Kit and loaded on SDS-PAGE followed by Western blotting probing for GFP using Rabbit anti-GFP (Ab290) and HRP conjugated goat anti-rabbit IgG (SantaCruz Biotechnology).

### In vivo crosslinking

Hundred milliliter of LB medium (with and without 350 mM of NaCl) containing Ampicillin (100 μg/ml) was inoculated with overnight grown cells at 0.1% inoculum for each strain. Cells were grown till they reach O.D_600_ 0.5 and harvested at 3150 × *g* for 10 min at RT. Pellet was finally resuspended in 2 ml of spent media and induced for 5 min at 37 °C with 0.5% arabinose for GFP expression. Cells were harvested and resuspended in 2 ml of 1X PBS containing protease inhibitor cocktail (Roche) and 500 μl of resuspended cells was taken as a control for uncrosslinked sample (referred to as -DSG in the text). To the remaining cells, 300 μM of Di (N-succinimidyl) glutarate (DSG) was added and incubated at 37 °C for 10 min for crosslinking. Reaction was quenched with 100 mM of Tris, pH 8 for 5–10 min at room temperature. Cells were harvested at 15,000 × *g*, 2 min at room temperature and lysed by freeze-thaw method in the presence of protease inhibitors. Thirty microgram of protein was loaded on SDS-PAGE followed by western blotting probing for GFP.

### Western blotting experiment

*E. coli* cells were inoculated in LB and grown at 37 °C for 5 h. Cells were harvested and resuspended in 1X PBS (Himedia; ML023). Equal volume of 2X lysis buffer was added and boiled at 95 °C for 15 min followed by centrifugation at 17,530 × *g* for 15 min. The supernatant was collected and estimated for the amount of protein. Thirty microgram of protein was used for SDS-PAGE and western blotting post-transferring onto nitrocellulose membrane (Millipore; HATF00010). The blots were decorated with antibodies against GroEL (Enzo; 9A1/2) and DnaK (Enzo; 9E2/2) isolated from mouse. Blots were developed using HRP conjugated Goat anti-mouse IgG (Genscript) and luminata crescendo (Millipore). Densitometric analysis was done using ImageJ. (Rasband, W.S., ImageJ, U.S. National Institutes of Health, Bethesda, Maryland, USA, http://imagej.nih.gov/ij/, 1997–2011).

### GFP refolding assay

Sixty micromolar native GFP (or mutant) was mixed with 8 M GuHCl prepared in GFP refolding buffer (Buffer-A) (Tris-Cl (25 mM), KCl (150 mM), MgCl_2_ (10 mM), DTT (2 mM), pH 7.4) in 1:3 ratio. After incubation at 25 °C for 1 h, refolding of GFP was initiated in Buffer-A by diluting the mixture 100-fold (final GFP concentration 150 nM). Real time fluorescence of GFP was monitored in Fluorolog 3 spectrophotometer (Horiba Jobin Yvon, with operating software FluorEssence v3.0) at 25 °C, with excitation wavelength 488 nm (2 nm slit width) and emission wavelength 515 nm (5 nm slit width), enabling ‘anti-photobleaching’ mode. The data acquired from refolding assays was analyzed in OriginPro 8 (OriginLabcorporation).

### Thermal melt

Buffer-A with or without 100 mM small molecules was added with purified GFP (Wt or mutant) to a final concentration of 150 nM. Twenty microliter of this whole mixture was aliquoted in a 386 well RT-PCR plate. The thermal melt was carried out in CFX384™ Real Time System attached to a C1000 Touch Thermal Cycler (Biorad). The program was set to go from 25 °C to 90 °C with a step of 1 °C and incubation time of 2 min/°C. The data were collected as GFP fluorescence at each step, and fit to a linear equation to obtain midpoint of each curve as melting temperature (Tm).

### Isolation of protein-free cell extract

*E. coli* K-12 (BW25113) strain was grown in 5 ml LB broth at 37 °C, 200 rpm orbital shaking for overnight and this primary culture was used to inoculate a 500 ml secondary culture in LB broth. After growing the secondary culture in identical conditions for 3 h (OD 0.7–0.8), the culture was centrifuged at 3150 × *g* for 30 min at 37 °C. Five milliliter of boiling hot ultrapure water was added directly to the pellet, resuspending it vigorously, and the cell suspension was immediately collected in a glass test tube. The glass tube was kept in a boiling water bath for 30 min, ensuring maximum lysis of cells and precipitation of proteins. The resulting suspension was cooled down to room temperature and was centrifuged at 15,000 × *g* for 20 min. Absence of protein in the supernatant, the protein-free cell extract, was confirmed by Bradford protein estimation assay.

### Simulation

Numerical simulations were performed using the ODEs defined in Fig. 4 to model a basic framework to check the apparent rate of degradation as a function of folding rate in vivo. The simulation was set up with 1000 μM S (pool of DNA/RNA that is competent to make proteins). S can form U (nascent polypeptides) with an overall unimolecular rate constant of ktrans (a simplistic combination of rates of transcription and translation). The pool of U can either convert to F (folded GFP) or be degraded with the unimolecular rate constants of k_f_ and k_deg_, respectively. The pool of S can be blocked by I (inhibitor of translation) with a rate constant of k_I_. This was included to mimic translation arrest by chloramphenicol. The simulation was run in the absence of I for 300 sec. Following this, 1 mM of I was dosed into the simulation to rapidly quench S. Following this we monitored the total concentration of uncleaved GFP (U + F) over time to mimic the results obtained from the chase experiments performed with anti-GFP antibodies.

### Conservation score and fractional ASA measurement through FoldX

PDB structures were used to calculate the conservation score of each of the residues using multiple sequence alignment through CONSURF^60^. Fractional ASA, and temperature factor of a residue was obtained using VADAR^61^. FoldX package was used to obtain the predicted stability value for each of the mutants^62^. The calculations were repeated at least three independent times to obtain an average and standard deviation value for the predicated stability values.

### Quantification and statistical analysis

Student’s *t*-test and R package for nonlinear regression was used for statistical analysis. Flow-cytometry data were analyzed using octave.

## Supplementary information

Supplementary Information

Peer Review File

## Data Availability

The Genome sequencing and transcriptomics data generated during the study are available at the NCBI BioProject database with accession SAMN14543567-SAMN14543577 [https://www.ncbi.nlm.nih.gov/bioproject/PRJNA623109]. All the other data supporting the findings of this study are available within the article and the Supplementary Information files. The source data underlying Figs. 2i, 3e, 4a–d, 5b, 5d–f, 6a, 7a, b, 8f, i and Supplementary Figures 1B, 1C, 3B, 3C, 3E–G, 4C, 4F, 5, 6B and 6C are provided in source data files. Source data are provided with this paper.

## Source data

Source Data
