# Supplementary material for: Distinct metabolic states of a cell guide alternate fates of mutational buffering through altered proteostasis
Source: Nat Commun. 2020 Jun 10;11:2926. doi: 10.1038/s41467-020-16804-6 (PMC7286901; doi:10.1038/s41467-020-16804-6)
Supplement: Supplementary file 1 — Supplementary Information [file 41467_2020_16804_MOESM1_ESM.pdf]

Distinct metabolic states of a cell guide alternate fates of mutational buffering through altered proteostasis

Verma et al.

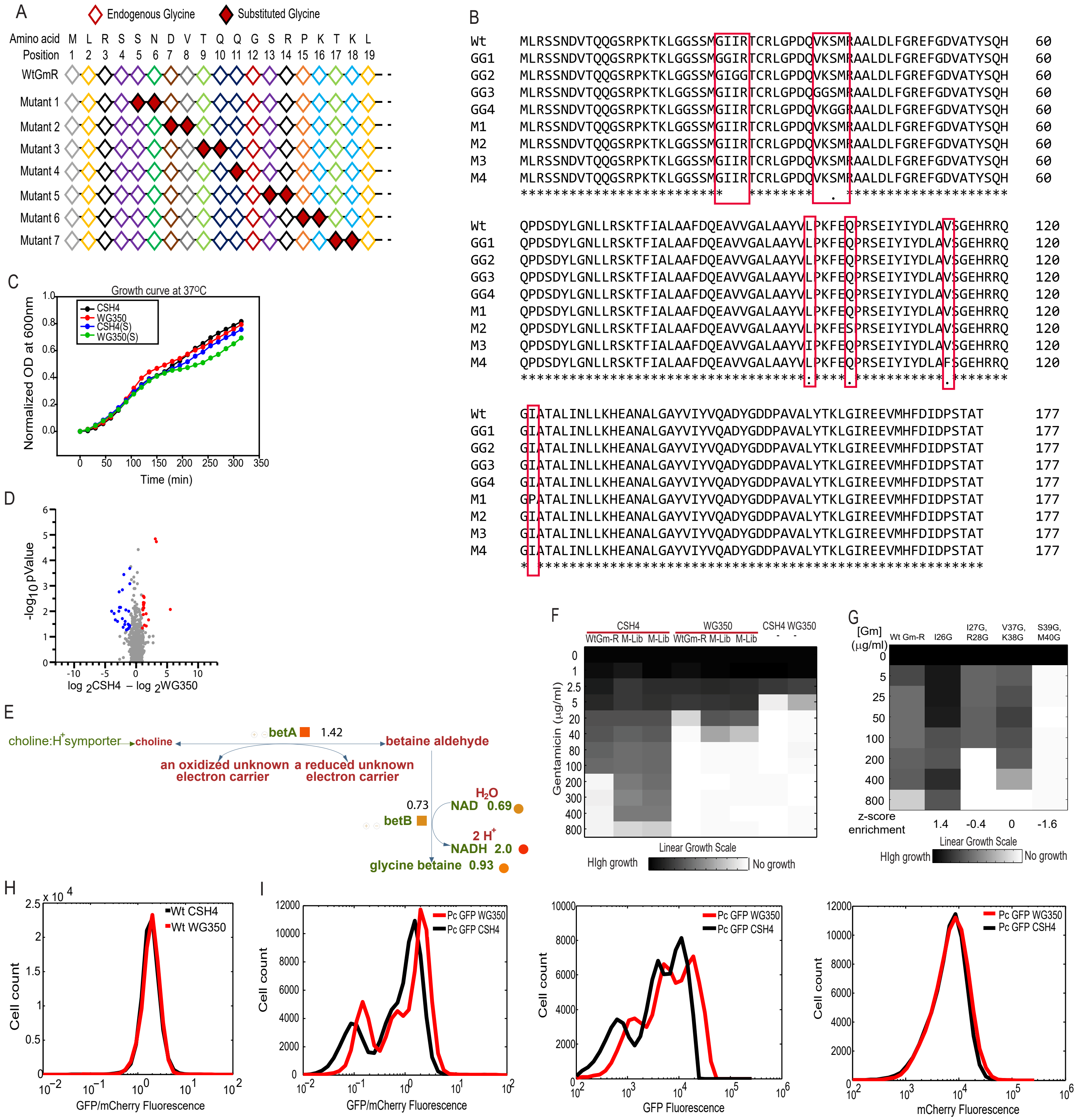

Supplementary Figure 1: Strain specific metabolic differences lead to differential mutation buffering capacity (Related to Figure 2)

A. Schematic of glycine doublet substitution library (GG) of Gm-R mutants where two consecutive amino acids have been substituted with two glycine residues (red solid filled) starting from 5th amino acid.

B. Amino acid sequence alignment of Wt and Gm-R mutants. Residues mutates are shown in red boxes.

C. Growth curve for *E. coli* strains CSH4 (black, blue) and WG350 (red, green) in presence and absence of 350 mM NaCl added in excess to the LB medium while growing at 37°C.

D. Scatter plot for mean of $\log_2$ of fold change in metabolite concentrations between CSH4 and WG350 against -log10 of p-value. The significantly altered metabolites (2-tailed paired Student's t-test p-value < 0.05, 5 biological replicates for each sample) are shown as colored dots**.**

E. Pathway for production of glycine betaine in *E. coli* showing correlation between metabolomics and transcriptomics. Metabolites upregulated are shown as circular dots and transcripts for the genes are shown as square dots. Color intensity corresponds to fold upregulation.

F. Growth of untransformed CSH4(-), WG350(-) and cells transformed with Wt Gm-R and glycine doublet mutant library plasmids (M-Lib) in increasing concentrations of Gentamicin (0-800 µg/ml). Increase in growth is shown as increase in color density.

G. Correlation between NGS based quantification of enrichment scores (z-score) and MIC based semi-quantitative activity measurement for Wt Gm-R and mutants I26G, I27G- R28G, V37G-K38G, S39G-K40G of glycine doublet mutant library.

H. Histogram for *in vivo* fluorescence of Wt GFP (represented as ratio of GFP/mCherry) in CSH4 (black) and WG350 (red).

I. Histograms for ratio of GFP/mCherry fluorescence (left) and independent fluorescence of GFP (middle) and mCherry channel (right) of mutant library Pc in CSH4 and WG350.

(Source data is provided as source data file Supplementary Figure 1)

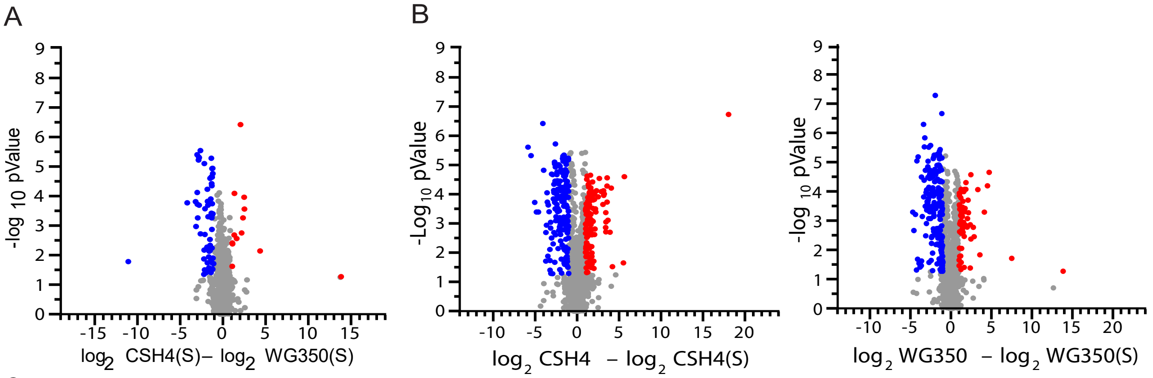

C

```
Wt   MSKGEELFTGVVPILVELDGDVNGHKFSVSGEGEGDATYGKLTLKFICTTGKLPVPWPTL   60
C1   MSKGEELFTGVVPILVELDGDVNCHKFSVSGEGEGDATYGKLTLKFICTTGKLPVPWPTL   60
C3   MSKGEELFTGVVPILVELDGDVNGHKFSVSGEGGGDATYGKLTLKFICTTGKLPVPWPTL   60
C6   MSKGEELFTGVVPILVELDGDVNGHKFSVSGEGEGDATYGKLTLKFICTTGKLPVPWPTL   60
     *********************** ********** *************************

Wt   VTTFGYGVQCFARYPDHMKQHDFFKSAMPEGYVQERTIFFKDDGNYKTRAEVKFEGDTLV   120
C1   VTTFGYGVQCFARYPDHMKQHDFFKSAMPEGYVQERTIFFKDDGNYKTRAEVKFEGDTLV   120
C3   VTTFGYGVQCFARYPDHMKQHDFFKSAMPEGYVQERTIFFKDDGNYKTRAEVKFEGDTLV   120
C6   VTTFGYGVQCFARYPDHMKQHDFFKSAMPEGYVQERTIFFKNDGNYKTRAEVKFEGDTLV   120
     *****************************************:******************

Wt   NRIELKGIDFKEDGNILGHKLEYNYNSHNVYIMADKQKNGIKVNFKIRHNIEDGSVQLAD   180
C1   NRIELKGIDFKEDGNILGHKLEYNYNSHNVYIMADKQKNGIKVNFKIRHNIEDGSVQLAD   180
C3   NRIELKGIDFKEDGNILGHKLEYNYNSHNVYIMADKQKKGIKVNFKIRHNIEDGSVQLAD   180
C6   NRIELKGIDFKEDGNILGHKLEYNYNSHNVYIMADKQKNGIKVNFKIRHNIEDGSVQLAD   180
     **************************************:*********************

Wt   HYQQNTPIGDGPVLLPDNHYLSTQSALSKDPNEKRDHMVLLEFVTAAGITHGMDELYK   238
C1   HYQQNTPIGDGPVLLPDNHYLSTQSALSKDPNEKRDHMVLLEFVTAAGITHGMDELYK   238
C3   HYQQNTPIGDGPVLLPDNHYLSTQSALSKDPNETRDHMVLLEFVTAAGITHGMDELYK   238
C6   HYQQNTPNGDGPVLLPDNHYLSTQSALSKDPNEKRDHMVLLEFVTAAGITHGMDELYK   238
     ******* *************************.************************
```

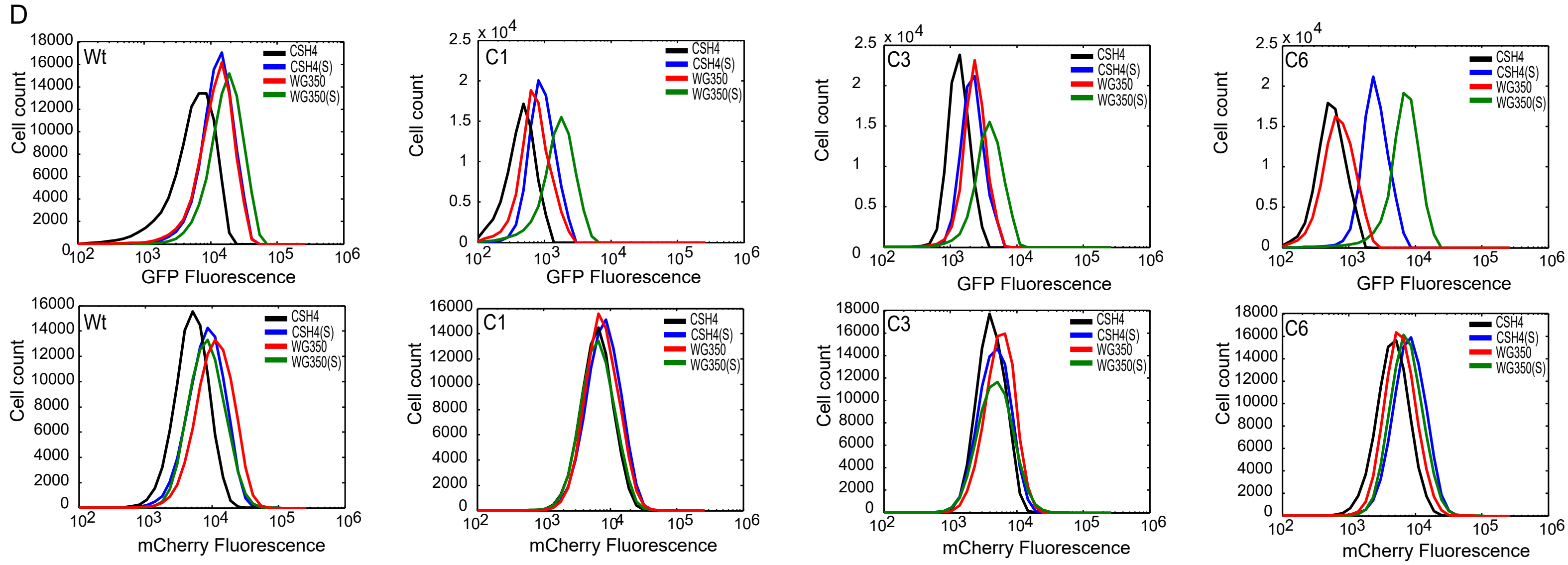

Supplementary Figure 2: Osmotic stress induced modification in cellular metabolism changes the subset of mutations buffered (Related to Figure 3)

A. Scatter plot for log2 of fold change in metabolic features between WG350 and CSH4 during osmotic shock against p-value. The significantly different metabolites obtained using 2-sided student's t test (p-value < 0.05, 5 biological replicates for each sample) are represented in colored dots.

B. Scatter plot for log2 of fold change in metabolic features within strains CSH4 (left) and WG350 (right) on osmotic shock(s) against p-value. The significantly different metabolites (p-value < 0.05, 5 biological replicates for each sample) are represented in colored dots.

C. Amino acid sequence alignment of Wt GFP (YeGFP) with the isolated mutants C1, C3 and C6. Mutations are shown in red boxes.

D. Histograms for GFP (top panel) and mCherry fluorescence (bottom panel) of the Isolated clones (Wt, C1, C3 and C6 GFP) in WG350 and CSH4 in the presence and absence of 350 mM NaCl.

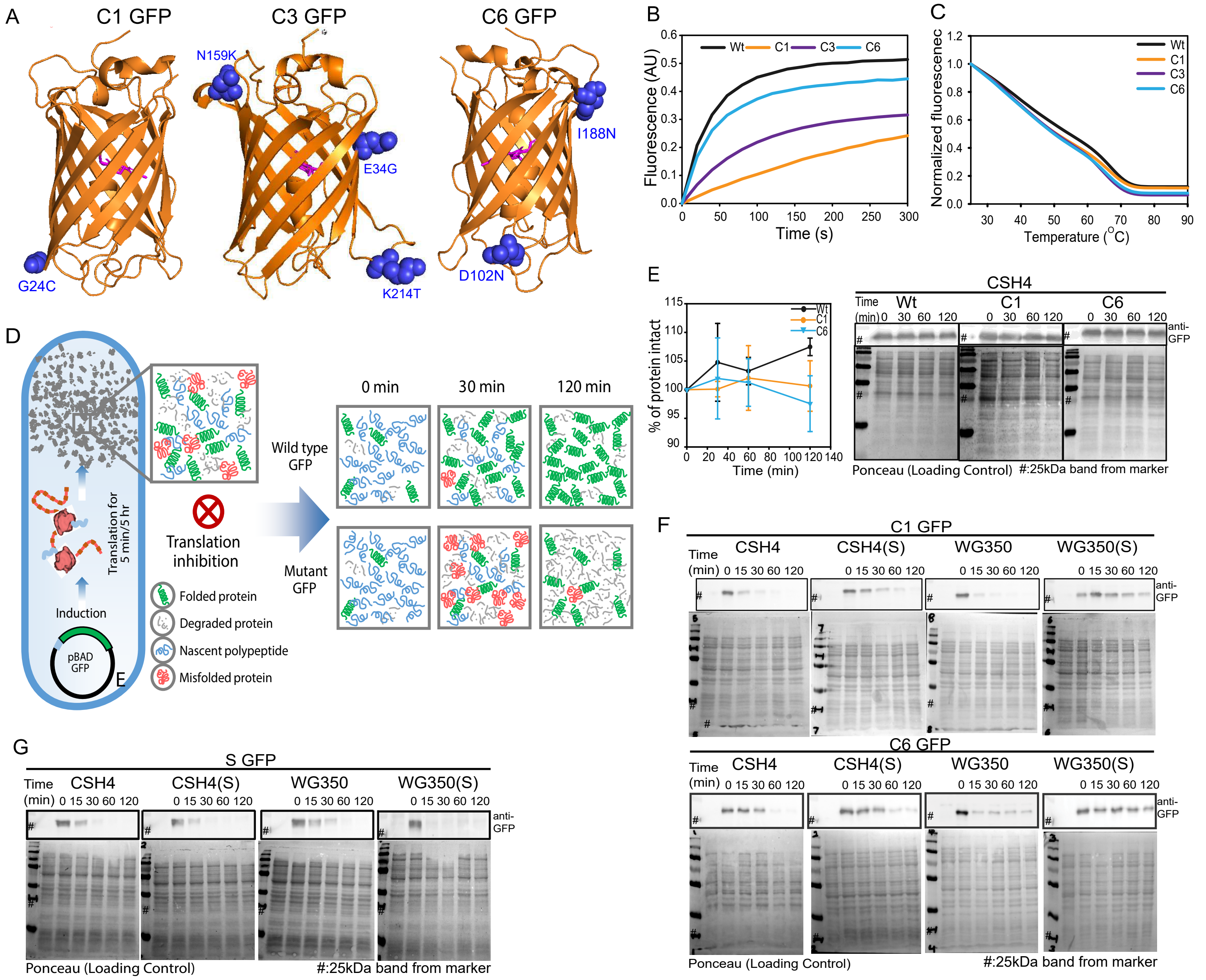

Supplementary Figure 3: Fluorescence increase is not due to better proteostasis in WG350(S) (Related to Figure 4)

A. Mutations buffered in WG350 while growing in 350 mM NaCl (C1, C3, and C6) are marked on the GFP crystal structure (PDB ID: 1GFL) (Yang, Moss and Phillips, 1996). Fluorophore at the centre of the barrel is highlighted in magenta.

B. Refolding traces of Wt and GFP mutants obtained on unfolding the proteins in 6 M GuHCl, followed by a 100-fold dilution into Buffer-A (50 mM Tris, 150 mM KCl, 10 mM $MgCl_2$, 2 mM DTT, pH 7.4) to a final concentration of 200 nM for the proteins. The GFP fluorescence (AU) is plotted against time (s).

C. Average thermal melt curves for Wt, C1, C3 and C6 GFP. Fluorescence of each mutants at different temperatures has been normalized to its respective fluorescence at 25°C.

D. Schematic of Chloramphenicol-based chase for degradation of GFP as described in Methods. After a short (5 min) induction of GFP with 0.5% arabinose, translation was stalled with Chloramphenicol. Cells were lysed at different points and the amount of intact GFP was monitored by immunoblotting with anti-GFP antibody.

E. Plot for the gel-based quantification of intact GFP (left) and representative images for immunoblotting to chase degradation of Wt, C1 and C6 GFP in CSH4 after 5 hr induction of GFP with 0.5% arabinose followed by translation arrest with Chloramphenicol. Ponceau has been used as loading control.

F. Representative images from 3 biological replicates for immunoblotting to chase degradation of nascent chains of C1 and C6 GFP as described in Methods in CSH4 and WG350 in presence and absence of osmotic stress. GFP degrades slowest in WG350(S). Ponceau has been used as loading control.

G. Representative images from 3 biological replicates for immunoblotting to chase degradation of sGFP in CSH4 and WG350 in presence and absence of osmotic stress. Ponceau has been used as loading control.

(Source data is provided as source data file Supplementary Figure 3)

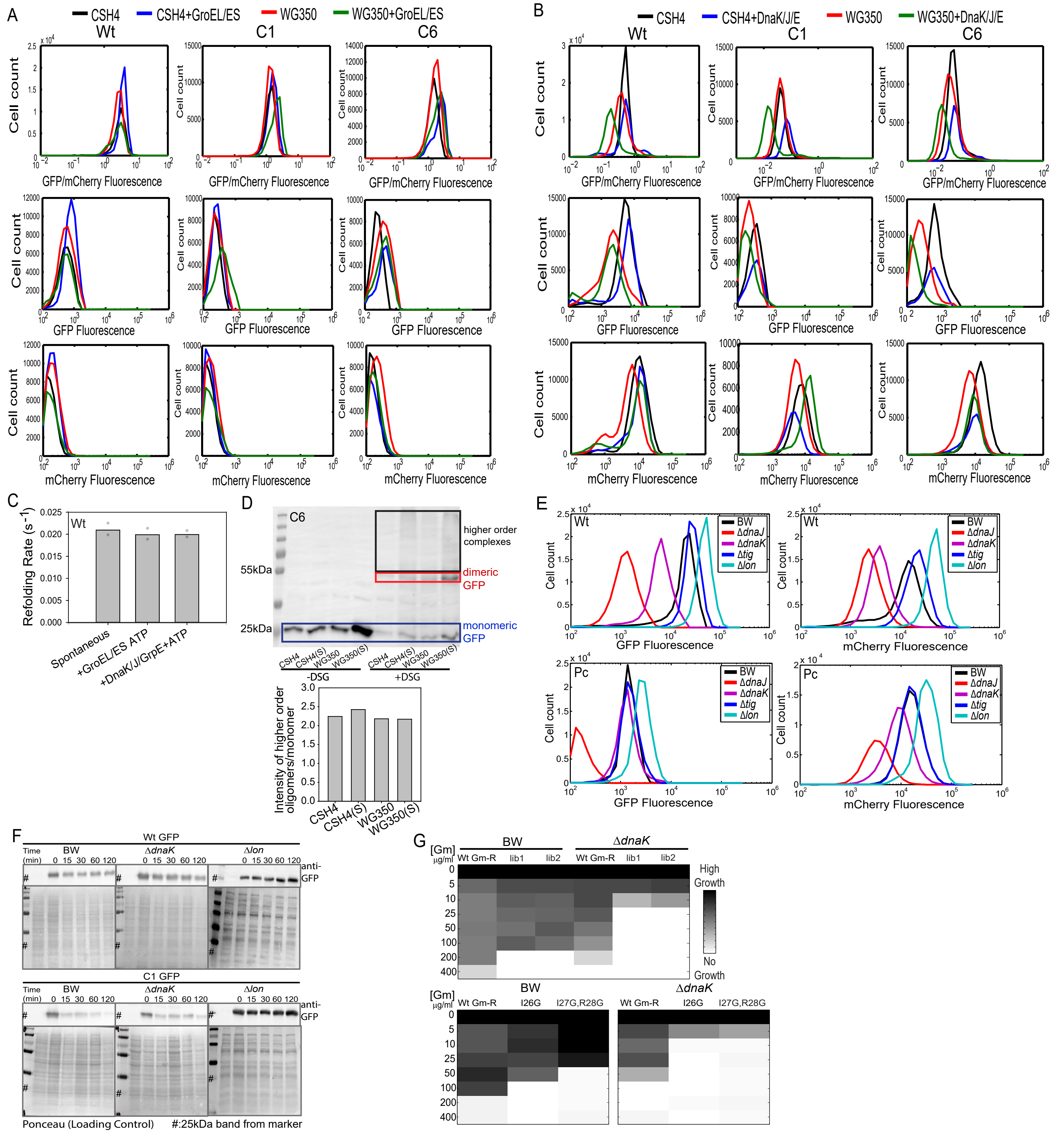

Supplementary Figure 4: Observed buffering of GFP mutants is not channelled only through molecular chaperones (Related to Figure 5)

A. Histograms for GFP/mCherry Fluorescence (top panel), GFP (middle panel) and mCherry fluorescence (bottom panel) in absence of overexpressed GroEL/ES (black, red, 2 biological replicates) and while co-expressed with GroEL/ES (blue, green, 2 biological replicates) in CSH4 and WG350. Induction protocol was followed as described in Methods.

B. Histograms for GFP/mCherry Fluorescence (top panel), GFP (middle panel) and mCherry fluorescence (bottom panel) in absence of overexpressed DnaK/J/GrpE (black, red, 2 biological replicates) and while co-expressed with DnaK/J/GrpE (blue, green, 2 biological replicates) in CSH4 and WG350.

C. Bar graph for mean rate of refolding for unfolded Wt GFP in presence and absence of 400 nM DnaK/800 nM DnaJ/400 nM GrpE or 400 nM GroEL/800 nM GroES.

D. Immunoblotting for GFP showing the recruitment of nascently formed C6 GFP into multimeric complexes. Free (or monomeric) GFP are shown with blue box, dimeric GFP are shown in red box and the multimeric complexes are shown in black box. The ratio of GFP in multimeric complex and the amount in monomeric state is shown in the accompanying bar graph.

E. Histograms of GFP or mCherry fluorescence of Wt GFP and pool of GFP mutants having compromised fluorescence (Pc) in BW and molecular chaperone and protease knockout strains (Δ*dnaJ,* Δ*dnaK,* Δ*tig,* Δ*lon*).

F. Representative images from 3 biological replicates for immunoblotting of chase degradation of Wt and C1 GFP in BW, chaperone (Δ*dnaK*), and protease (Δ*lon*) knockout strain. Ponceau has been used as loading control.

G. Heatmaps for growth-based activity of Wt and Gm-R mutant library (lib1, lib2) (top panel), Wt Gm-R and Gm-R mutants (I26G, I27G-R28G) in BW and molecular chaperone knockout strain (Δ*dnaK*) in increasing concentrations of Gentamicin.

(Source data is provided as source data file Supplementary Figure 4)

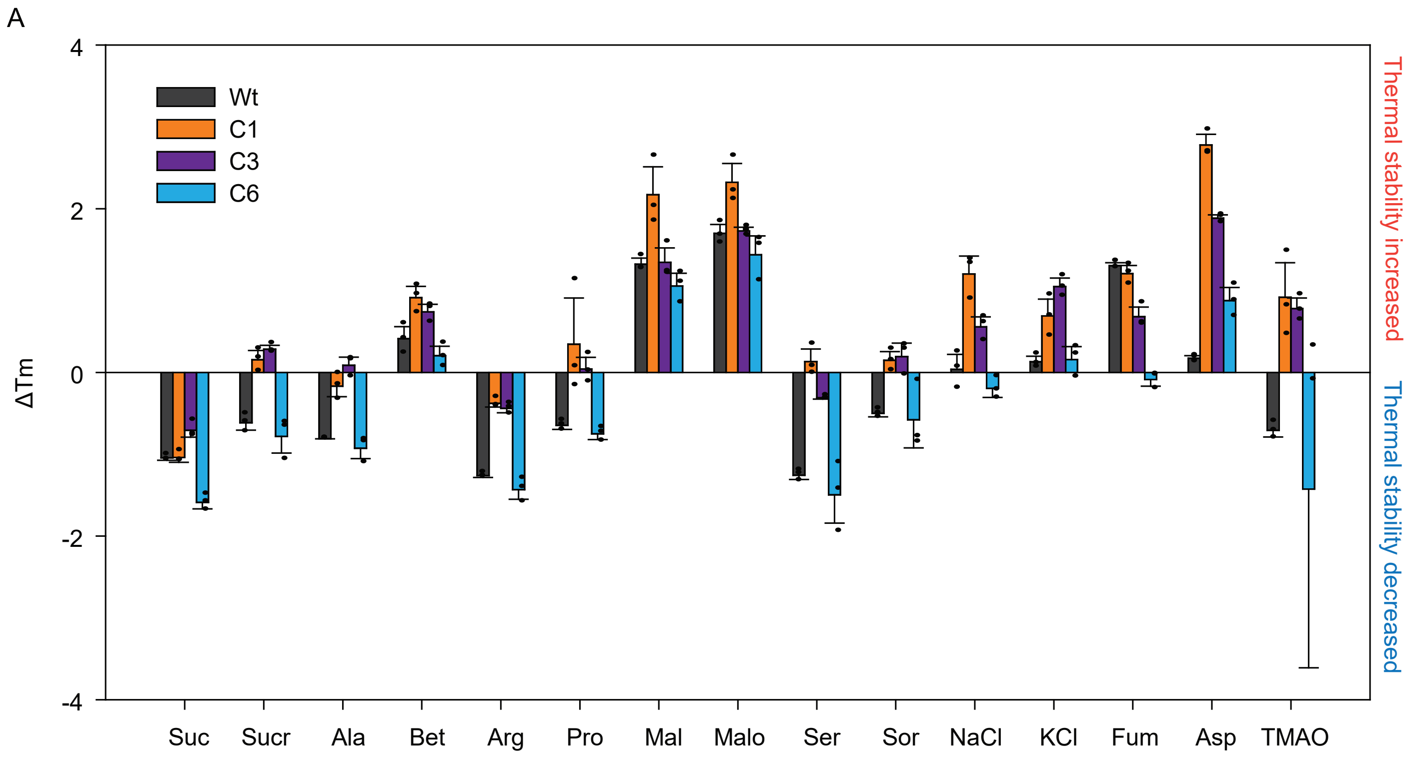

Supplementary Figure 5: Different small molecules affect energetics of folding in a mutant specific manner (Related to Figure 6)

A. Thermal melting profile of Wt, C1, C3 and C6 GFP as mean of change in melting temperature (ΔTm) in presence of different small molecules. The mean of change in Tm in presence of a small molecule is obtained by subtracting Tm in presence of Buffer-A (50 mM Tris, 150 mM KCl, 10 mM MgCl2, 2 mM DTT, pH 7.4) from Tm in presence of that respective small molecule (100 mM). Error bars indicate standard deviation among 3 technical replicates.

Suc: Succinate, Sucr: Sucrose, Ala: Alanine, Bet: Betaine, Arg: Arginine, Pro: Proline, Mal: Malate, Malo: Malonate, Ser: Serine, Sor: Sorbitol, Fum: Fumarate, Asp: Aspartate, TMAO: Trimethylamine-N-oxide

(Source data is provided as source data file Supplementary Figure 5)

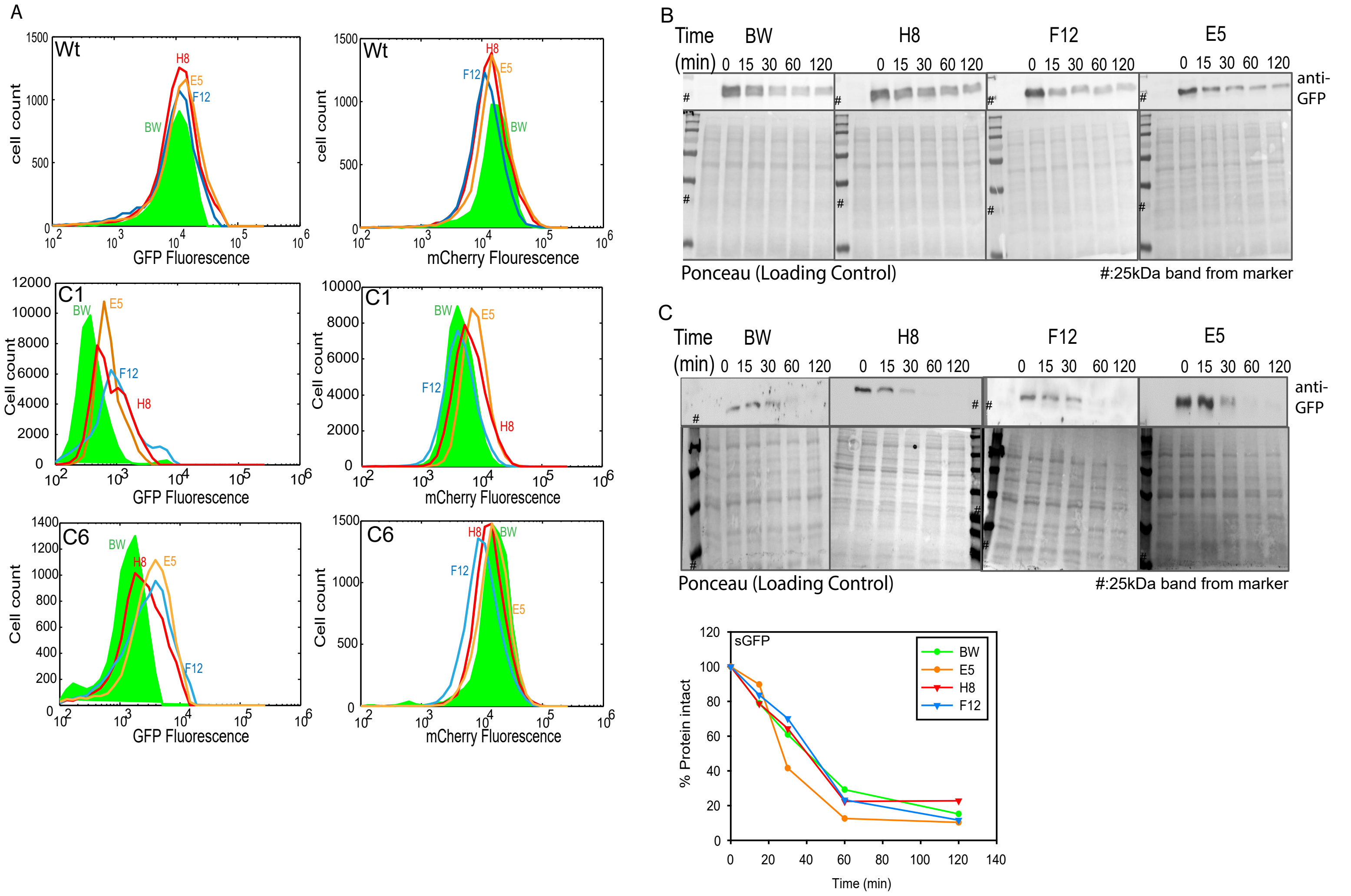

Supplementary Figure 6: Modification in metabolome due to evolution changes the mutational buffering capacity of the cell (Related to Figure 8)

A. Histograms for fluorescence of Wt and GFP mutants C1 and C6 represented as GFP (left panel) and mCherry fluorescence (right panel) in unevolved BW (WT *E. coli* K12, BW25113) and evolved osmotolerant strains E5, F12, H8.

B. Representative images from 3 biological replicates of Chloramphenicol based chase for checking degradation rates of C1 GFP in BW (WT *E. coli* K12, BW25113) and evolved strains E5, F12, H8. GFP is degraded slower in evolved strains than BW.

C. Chloramphenicol based chase for sGFP in BW (WT *E. coli* K12, BW25113) and evolved strains E5, F12, H8. sGFP is degraded with similar rates in evolved and unevolved BW as shown in densitometric analysis plot at the bottom.

(Source data is provided as source data file Supplementary Figure 6)

Supplementary Table 1: Key resources table

REAGENT	SOURCE	IDENTIFIER
**Antibodies**		
Anti GroEL (*E.coli*) mouse monoclonal	ENZO	9A1/2
Anti DnaK (*E.coli*) mouse monoclonal	ENZO	8E2/2
Anti DnaJ (*E.coli*) rabbit polyclonal	ENZO	SPA410
Anti Tig (*E.coli*) rabbit polyclonal	Genescript	A01329
Anti GFP rabbit polyclonal	Abcam	Ab290
Anti RecA rabbit polyclonal	Abcam	Ab125096
Anti Mouse secondary	Santa Cruz Biotechnology	SC2005
Anti Rabbit secondary	Santa Cruz Biotechnology	SC2030
Di(N-succinimidyl) glutarate (DSG)	Sigma	80424
**Bacterial and Virus Strains**		
E.coli BL21 (DE3)		
E.coli DH5α		
BW (*E.coli* K12, BW25113)<br>F-, DE(araD-araB)567, lacZ4787(del)::rrnB-3, LAM-, rph-1, DE(rhaD-rhaB)568, hsdR514	(Baba *et al.*, 2006)	CGSC#: 7636
Δ*Tig* (JW0426-1)<br>*F-, Δ(araD-araB)567, ΔlacZ4787(::rrnB-3), Δtig-722::kan, λ-, rph-1, Δ(rhaD-rhaB)568, hsdR514*	(Baba *et al.*, 2006)	CGSC#: 8589
Δ*dnaK (*JW0013-4)<br>F-, Δ(araD-araB)567, ΔlacZ4787(::rrnB-3), λ-, rph-1, ΔdnaK734::kan, Δ(rhaD-rhaB)568, hsdR514	(Baba *et al.*, 2006)	CGSC#: 8342
Δ*dnaJ (*JW0014-1)<br>F-, Δ(araD-araB)567, ΔlacZ4787(::rrnB-3), λ-,rph-1, ΔdnaJ735::kan,Δ(rhaD-rhaB)568, hsdR514	(Baba *et al.*, 2006)	CGSC#: 8343
ΔclpA<br>F-, Δ(araD-araB)567, ΔlacZ4787(::rrnB-3), λ-, ΔclpA783::kan,rph-1, Δ(rhaD-rhaB)568, hsdR514	(Baba *et al.*, 2006)	CGSC#: 8898
ΔclpP<br>F-, Δ(araD-araB)567, ΔlacZ4787(::rrnB-3), ΔclpP723::kan, λ-,rph-1, Δ(rhaD-rhaB)568, hsdR514	(Baba *et al.*, 2006)	CGSC#: 8590
ΔclpX<br>F-, Δ(araD-araB)567, ΔlacZ4787(::rrnB-3), ΔclpX724::kan, λ-, rph-1,Δ(rhaD-rhaB)568, hsdR514	(Baba *et al.*, 2006)	CGSC#: 8591
ΔhslU<br>F-, Δ(araD-araB)567, ΔlacZ4787(::rrnB-3), λ-, rph-1, Δ(rhaD-rhaB)568, ΔhslU790::kan, hsdR514	(Baba *et al.*, 2006)	CGSC#: 10817

ΔhslV<br>F-, Δ(araD-araB)567, ΔlacZ4787(::rrnB-3), λ-, rph-1, Δ(rhaD-rhaB)568, ΔhslV720::kan, hsdR514	(Baba *et al.*, 2006)	CGSC#: 10818
ΔsecB<br>F-, Δ(araD-araB)567, ΔlacZ4787(::rrnB-3), λ-, ΔsecB721::kan, rph-1, Δ(rhaD-rhaB)568, hsdR514	(Baba *et al.*, 2006)	CGSC#: 10640
CSH4<br>F, lacZ1125, λtrpA49(Am), relA1, rpsL150(strR), spoT	(Newton *et al.*, 1965)	CGSC#: 8196
WG350<br>F-, lacZ1125, λ^-, Δ(putA-putP)101, trpA49(Am), ΔproU-600, relA1, rpsL150(strR), spoT1, Δ(proP-melB)212	(Culham *et al.*, 1993)	CGSC#: 8195
**Oligonucleotides**		
GFP Forward primer (to clone in pET SUMO)<br>CCCGGATCCATGTCTAAAGGTGAAGAATTATTCACTGGTGTTGTCCC	This Paper	
GFP Reverse primer (to clone in pET SUMO)<br>CCCAAGCTTTTATTTGTACAATTCATCCATACCATGGGTAATA	This Paper	
GFP Forward primer (to clone in pBAD vector)<br>TGTGCTGAATTCACATATATGTCTAAAGGTGAAGAATTATTCA	This Paper	
GFP Reverse primer (to clone in pBAD vector)<br>ACGGCCAAGCTTTTATTTGTACAATTCATCCATACCATGGG	This Paper	
RBS mCherry Forward primer (to clone in pBAD vector)<br>ACAAAGCTTTCCATACCCGTTTTTTGGGCTAACAGGAGGAATTAACCATGGTGA	This Paper	
RBS mCherry Reverse primer (to clone in pBAD vector)<br>TCTAAGCTTCTACTTGTACAGCTCGTC	This Paper	
**Recombinant DNA**		
pOFX ELES (pOFX tac-SL2)	(Castanié *et al.*, 1997)[70]	
pOFX DnaK/J/GrpE (pOFX tac-KJE1)	(Castanié *et al.*, 1997)[70]	
pET duet1 dnaK	(Tiwari *et al.*, 2013)[71]	
pET duet1 dnaJ	(Tiwari *et al.*, 2013)[71]	
pET duet1 grpE	(Tiwari *et al.*, 2013)[71]	
pET duet1 Mge1	(Tiwari *et al.*, 2013)[71]	
pET SUMO sGFP	Sadat & Mapa (in process)	
pBAD Gm-R	(Bandyopadhyay *et al.*, 2012)[25]	
pBAD I26G Gm-R	This Paper	

pBAD I27G, R28G Gm-R	This Paper	
pBAD 37G Gm-R	This Paper	
pBAD 39G Gm-R	This Paper	
pET SUMO Wt GFP	This Paper	
pET SUMO C1GFP	This Paper	
pET SUMO C3FP	This Paper	
pET SUMO C6GFP	This Paper	
pBAD Wt GFP mCherry	This Paper	
pBAD C1GFP mCherry	This Paper	
pBAD C3GFP mCherry	This Paper	
pBAD C6GFP mCherry	This Paper	
**Software and Algorithms**		
Chimera		
Proteowizard	(Brown *et al.*, 2005)[72]	
XCMS	(Smith *et al.*, 2006; Silva *et al.*, 2014)[73,74]	
Maven	(Lu *et al.*, 2010; Clasquin, Melamud and Rabinowitz, 2012) [65,66]	
FastQC	http://www.citeulike.org/user/nailest/author/Andrews:S	
Trimmomatic	(Bolger, Lohse and Usadel, 2014)[61]	
Kallisto	(Zhang *et al.*, 2017; Bray *et al.*, 2016)[62,63]	
EBseq	(Leng *et al.*, 2013)[64]	
ImageJ		
Origin Pro8		

Supplementary Table 2: List of metabolites along with their log_2 of fold change and p-values obtained using 2-tailed paired student's t test from untargeted metabolite profiling and analysis using MAVEN platform (Related to Figure 2 and 3)

S.no.	med Mz	med Rt	Max Quality	compound	Log_2 fold change				$-Log_{10}$ p-value			
					CSH4 - WG350	CSH4 - CSH4 (S)	WG350 - WG350 (S)	CSH4 (S)- WG350 (S)	CSH4-WG350	CSH4-CSH4 (S)	WG350 - WG350 (S)	CSH4 (S)- WG350 (S)
1	363.0344	2.041682	0.848391	N-Acetylputrescine	0.00	-14.03	-14.29	-0.26	0.01	0.97	1.54	0.45
2	283.0684	4.029608	0.849596	Geranyl-PP	0.00	-13.99	-13.89	0.10	0.05	1.67	1.43	0.20
3	151.025	1.858412	0.819399	fructose-1-6-bisphosphate	0.00	-12.65	0.00	12.65	0.00	2.24	0.43	5.16

4	116.070 5	2.341 379	0.828 605	succinate	0.00	-11.67	-25.78	-14.11	0.43	1.60	2.15	1.80
5	243.062 2	2.131 804	0.848 934	UMP	2.10	-4.63	-4.86	1.87	0.49	2.56	1.80	1.80
6	167.020 4	2.374 183	0.847 362	orotidine-5--phosphate	10.98	-4.25	-14.62	0.61	1.12	4.78	1.71	1.38
7	323.029 1	3.537 884	0.851 401	betaine	-0.93	-4.17	-7.94	-4.70	0.53	0.95	0.98	1.78
8	606.074 8	4.036 123	0.851 434	Octoluse Bisphosph ate	1.06	-3.88	-4.69	0.24	0.15	1.00	1.04	0.28
9	565.048 2	4.056 136	0.857 477	tryptophan	0.44	-3.75	-4.18	0.01	0.05	1.93	1.71	0.22
10	402.994 9	2.973 083	0.849 58	thiamine-phosphate	0.12	-3.73	-2.99	0.87	0.01	1.34	1.15	3.48
11	180.065 7	2.219 842	0.858 241	fumarate	-2.93	-3.27	-6.82	-6.49	0.88	0.51	3.44	1.03
12	203.081 9	1.413 762	0.840 261	FMN	1.54	-2.86	-1.06	3.34	1.17	1.98	0.65	4.25
13	203.081 9	1.505 575	0.858 171	asparagine	-1.04	-2.73	0.10	1.79	0.62	2.12	0.10	3.83
14	341.108 9	2.038 149	0.846 411	glutathione disulfide	-1.96	-2.52	-0.13	0.43	1.29	1.15	0.39	0.24
15	421.075 6	1.887 725	0.851 835	CMP	-1.08	-2.37	1.97	3.26	0.47	1.60	1.10	1.82
16	241.082 7	2.019 653	0.856 852	GMP	1.22	-2.33	-2.11	1.44	1.97	4.11	2.86	3.62
17	344.069 1	2.006 949	0.846 896	UDP	-0.13	-2.28	-1.68	0.47	0.10	4.33	3.04	0.73
18	424.039	1.499 295	0.860 667	aspartate	-0.40	-1.99	2.16	3.75	0.64	3.32	2.82	5.07
19	264.104 5	1.446 013	0.858 174	trehalose/s ucrose	1.55	-1.89	-1.60	1.84	3.11	3.67	2.71	3.08
20	866.124 9	1.450 47	0.857 708	Cellobiose	1.55	-1.89	-1.60	1.84	3.11	3.67	2.71	3.08
21	117.018 6	2.093 047	0.854 168	N-Acetyl-L-alanine	-1.80	-1.77	0.08	0.05	0.78	1.01	0.38	0.06
22	171.006 5	3.843 684	0.849 186	IMP	-0.41	-1.66	-1.07	0.18	0.38	2.09	1.54	0.45
23	253.009 9	2.067 247	0.861 783	Pyrogluta mic acid	-1.85	-1.53	-0.12	-0.43	0.64	0.67	0.41	0.80
24	368.998 9	3.985 863	0.849 202	S-methyl-5--thioadenos ine	-0.27	-1.28	-1.28	-0.28	0.15	1.34	1.39	0.43
25	266.070 7	1.685 908	0.833 351	ribose-phosphate	-0.60	-1.15	0.82	1.37	0.75	1.65	1.10	2.80
26	296.080 6	2.066 397	0.852 619	N-acetyl-L-ornithine	0.30	-1.10	-1.11	0.30	0.35	2.75	2.67	0.64

27	383.1152	3.042196	0.771733	Acetyllysine	0.05	-0.96	-0.98	0.04	0.11	1.80	2.12	0.13
28	229.0112	2.020989	0.85325	adenosine	0.89	-0.91	-1.32	0.48	0.93	2.14	2.15	2.26
29	375.131	2.323842	0.84781	xanthosine-5-phosphate	-0.18	-0.89	-0.73	-0.01	0.04	1.82	2.08	0.03
30	176.935	4.759889	0.852717	2-Isopropylmalic acid	0.23	-0.74	-0.66	0.30	0.24	1.32	1.66	0.68
31	128.0341	1.83335	0.853449	dCDP	-0.36	-0.47	0.46	0.58	0.07	1.29	0.49	2.60
32	168.0656	2.160493	0.853901	xanthosine	0.45	-0.45	-0.52	0.37	1.07	1.80	1.32	1.07
33	114.0551	10.95572	0.73141	p-hydroxybenzoate	-0.33	-0.42	-0.22	-0.14	0.67	0.25	1.34	0.49
34	225.0392	11.50664	0.84889	Sedoheptoluse bisphosphate	-0.26	-0.24	-0.07	-0.09	0.28	0.00	0.14	0.08
35	145.0301	1.69376	0.833608	cystathionine	0.19	-0.23	-1.06	-0.64	0.54	0.38	1.94	0.55
36	218.1029	1.981234	0.860063	hypoxanthine	-0.22	-0.19	-0.01	-0.04	0.52	0.74	0.11	0.03
37	137.0232	1.572158	0.857927	glutamine	-0.07	-0.16	-0.09	0.00	0.28	0.01	0.07	0.06
38	367.0198	1.393888	0.84899	UDP-D-glucose	-0.14	-0.14	-0.10	-0.09	0.64	0.59	0.14	0.02
39	131.0814	6.98723	0.856877	Kynurenine	0.22	-0.11	-0.34	-0.02	0.22	0.36	1.13	0.16
40	399.0131	5.573004	0.850927	riboflavin	-0.10	-0.09	-0.13	-0.14	0.07	0.04	0.50	0.29
41	319.0463	5.199954	0.849735	Thiamine pyrophosphate	-0.03	-0.09	-0.05	0.01	0.12	0.54	0.56	0.12
42	146.0447	5.07694	0.840613	Octoluse 8/1P	0.10	-0.08	-0.32	-0.14	0.20	0.50	0.53	0.25
43	742.0695	3.301201	0.735053	valine	0.22	-0.08	-0.36	-0.06	0.27	0.36	1.11	0.22
44	664.118	1.939427	0.662212	cyclic-AMP	0.27	-0.02	0.62	0.91	0.55	0.64	1.54	1.10
45	662.1027	8.18236	0.852802	tryptophan	0.28	-0.01	-0.40	-0.11	0.20	0.13	0.99	0.61
46	175.0351	1.743987	0.85161	glucose-6-phosphate	0.35	0.04	1.96	2.27	0.75	0.07	0.43	3.75
47	129.1023	6.07219	0.850308	thiamine	0.00	0.05	-0.08	-0.14	0.13	0.05	0.50	0.34
48	173.0922	2.708844	0.81459	pantothenate	0.40	0.06	-0.53	-0.18	0.33	0.03	1.38	0.97
49	130.0498	5.998872	0.854558	biotin	0.14	0.06	-0.17	-0.09	0.19	0.08	0.77	0.16

50	187.0716	1.777959	0.832413	shikimate-3-phosphate	0.04	0.06	0.20	0.18	0.44	0.47	1.26	0.37
51	188.0556	1.504867	0.772704	N-acetyl-glutamine	0.04	0.09	0.16	0.10	0.02	0.01	1.50	1.00
52	300.0466	1.719046	0.848407	guanine	-0.13	0.13	0.24	-0.02	0.45	0.03	0.79	0.22
53	179.0552	1.501844	0.85144	inosine	-0.21	0.17	1.76	1.37	0.00	0.07	2.49	1.18
54	134.0294	2.00565	0.855251	dGMP	0.40	0.25	0.12	0.27	0.68	0.33	0.71	0.24
55	148.0426	3.28745	0.850251	D-sedoheptulose-1/7-phosphate	0.19	0.26	0.12	0.05	0.03	0.17	0.99	0.45
56	133.0131	1.936306	0.677158	methionine	-0.17	0.29	0.50	0.05	0.16	1.54	0.85	0.13
57	145.0971	1.390545	0.850053	UDP-N-acetyl-glucosamine	-0.21	0.40	0.18	-0.42	0.17	0.40	0.66	0.17
58	205.0347	2.536442	0.851966	proline	-0.88	0.40	-1.20	-2.48	0.33	0.24	0.81	1.84
59	289.1155	1.833144	0.851898	a-ketoglutarate	0.38	0.42	1.06	1.02	1.23	1.41	2.15	1.78
60	207.077	2.012048	0.833829	tyrosine	0.15	0.48	0.51	0.18	0.70	2.96	2.09	1.13
61	267.0725	1.519962	0.766428	Aminoadipic acid	0.22	0.49	0.01	-0.26	0.38	1.51	0.22	1.52
62	347.0395	2.04751	0.799432	N-acetyl-glutamate	0.26	0.53	0.47	0.19	0.21	1.07	0.92	0.36
63	135.0301	3.948531	0.850648	acetyl-CoA	0.12	0.55	1.77	1.34	0.04	2.12	2.55	2.32
64	112.4286	1.409344	0.858314	arginine	0.12	0.56	0.53	0.09	0.51	1.82	2.03	0.09
65	154.0611	1.914433	0.856739	glutathione	0.05	0.57	0.97	0.45	0.03	1.40	2.23	1.89
66	282.0861	1.459852	0.857308	ornithine	-0.04	0.58	0.55	-0.07	0.34	0.41	2.33	0.09
67	150.0411	2.037107	0.853763	thymidine	0.18	0.59	0.36	-0.04	0.44	1.94	2.04	0.31
8	362.0511	1.499073	0.851382	L-arginino-succinate	-0.38	0.63	2.28	1.27	0.38	1.49	2.82	0.84
69	611.1408	2.658341	0.853422	N-acetyl-glucosamine-1/6-phosphate	0.02	0.67	0.51	-0.14	0.21	2.95	2.74	0.61
70	306.0765	1.728165	0.823201	N-carbamoyl-L-aspartate	0.61	0.73	0.23	0.11	0.87	0.40	0.17	0.02
71	145.0607	2.06878	0.715853	uridine	0.12	0.75	0.56	-0.06	0.21	2.06	1.65	0.42

72	259.022	1.509566	0.783648	pyridoxine	-0.08	0.76	0.58	-0.26	0.11	3.64	3.39	2.73
73	178.0714	1.853829	0.854485	sn-glycerol-3-phosphate	0.08	0.83	0.36	-0.39	0.00	2.49	1.50	1.29
74	177.0395	2.04771	0.750985	xanthine	-0.02	0.84	0.62	-0.24	0.23	2.54	2.09	1.04
75	313.0589	2.024257	0.820586	citrate/isocitrate	0.48	0.85	0.79	0.42	0.68	1.42	1.53	1.24
76	109.5264	1.386311	0.855701	adenosine 5--phosphosulfate	0.31	0.85	0.50	-0.04	0.53	2.48	2.30	0.33
77	338.989	1.485925	0.818313	O-acetyl-L-serine	-0.14	0.93	0.85	-0.22	0.73	3.92	3.35	1.01
78	455.1014	6.339029	0.859511	2_3-dihydroxybenzoic acid	-1.45	0.94	-0.60	-2.99	2.03	1.58	1.11	3.67
79	784.1508	1.505536	0.850999	cytidine	-0.05	0.96	0.86	-0.15	0.01	4.59	3.03	0.30
80	321.0496	1.780281	0.860946	malate	-0.10	0.99	0.15	-0.95	0.12	1.90	0.05	4.07
81	346.0558	1.441355	0.856271	lysine	1.42	1.02	-0.06	0.34	1.83	1.76	0.14	1.05
82	686.1423	1.567711	0.853065	lipoate	0.04	1.08	0.61	-0.43	0.38	3.09	2.51	2.90
83	251.0776	4.043802	0.84958	FAD	-0.14	1.11	0.56	-0.69	0.23	3.17	0.87	1.06
84	250.0937	2.100759	0.846352	NADP+	0.30	1.11	1.08	0.27	0.04	0.65	1.58	0.25
85	386.0172	1.486119	0.856649	D-gluconate	-0.02	1.15	1.23	0.05	0.30	2.63	3.13	0.20
86	289.0352	1.507047	0.851621	2-dehydro-D-gluconate	-0.21	1.28	1.75	0.27	0.04	2.48	1.42	0.27
87	258.0375	1.453563	0.828056	histidine	0.30	1.30	1.00	0.01	0.46	2.73	3.55	0.11
88	195.0502	2.024109	0.852796	dTMP	-0.94	1.31	1.59	-0.66	1.19	1.38	2.20	1.03
89	199.0007	1.550444	0.856554	homoserine	-2.41	1.32	-1.46	-5.18	0.76	0.25	0.97	1.45
90	242.078	2.027355	0.788688	glucono-Î´-lactone	0.47	1.33	1.05	0.18	0.65	2.37	1.75	0.20
91	239.0147	1.507822	0.829343	myo-inositol	-0.17	1.38	1.11	-0.44	0.91	4.50	3.63	1.23
92	221.0598	1.489268	0.854986	citrulline	-0.20	1.46	1.22	-0.43	0.35	3.66	2.77	1.07
93	328.0454	3.954113	0.838432	succinyl-CoA/methylmalonyl-CoA	-0.26	1.66	1.51	-0.42	0.34	1.03	2.36	0.81
94	689.0877	2.038819	0.852447	cyclic bis (3-->5-) dimeric GMP	-0.18	1.68	1.61	-0.24	2.83	3.02	2.29	0.14

95	766.1091	1.525817	0.835367	Phenylpropiolic acid	0.14	1.75	1.23	-0.37	0.00	0.58	1.85	0.47
96	322.0445	3.260484	0.848054	coenzyme A	-1.70	1.77	1.48	-1.99	1.48	2.14	1.76	3.87
97	174.0874	1.793035	0.851756	D-erythrose-4-phosphate	0.19	1.89	1.83	0.14	0.42	1.66	1.94	0.01
98	191.019	1.979981	0.851608	Methylcysteine	0.95	2.07	1.70	0.58	0.58	1.09	0.71	0.54
99	341.1089	1.499742	0.852927	S-ribosyl-L-homocysteine-nega	0.12	2.14	0.40	-1.63	0.11	2.81	0.72	1.16
100	243.0804	1.504422	0.852072	deoxyinosine	0.31	2.21	1.33	-0.57	0.79	2.89	2.21	0.13
101	116.0711	1.985581	0.85367	NADH	-2.59	2.54	3.62	-1.52	1.44	0.68	3.51	0.63
102	132.0291	1.759809	0.850709	trehalose-6-Phosphate	2.73	2.56	0.30	0.47	3.62	3.26	0.95	2.36
103	131.045	1.46988	0.853972	deoxyadenosine	0.33	2.69	1.93	-0.43	0.47	4.44	2.27	0.21
104	173.1035	1.516454	0.850518	D-glucosamine-6-phosphate	0.68	2.77	1.61	-0.49	0.69	1.86	0.67	0.30
105	160.0605	1.466613	0.852018	glucosamine	0.42	2.83	2.92	0.51	0.89	3.49	2.96	0.18
106	426.0121	2.080937	0.848104	NAD+	-0.69	3.58	2.11	-2.15	0.07	2.46	0.79	1.78
107	266.091	1.819532	0.852153	Pyrophosphate	0.22	4.02	3.37	-0.43	0.55	5.90	4.13	0.82
108	187.108	1.889188	0.850813	Uric acid	-0.31	4.32	3.55	-1.08	0.17	4.38	2.87	0.74
109	808.1196	2.336574	0.830013	dephospho-CoA	-1.36	11.89	2.10	-11.15	1.16	1.83	1.11	0.44
110	145.0132	2.367599	0.838086	Cystine	1.04	12.24	11.20	0.00	2.09	7.53	1.86	0.43
111	153.0182	1.50954	0.803857	S-adenosyl L-homoCysteine	-0.86	12.98	13.85	0.00	1.67	8.01	6.35	#DIV/0!
112	175.0602	1.598831	0.850469	prephenate	0.46	13.61	13.14	0.00	0.07	5.34	1.52	0.75
113	193.0346	1.51109	0.831777	guanosine	0.08	14.42	1.34	-13.00	0.45	1.36	2.89	1.14

Key: med Mz: Median *m/z* for metabolites, medRt: Median Retention Time, Max Quality: quality of peaks detected, compound: Metabolite, CSH4 - WG350: log_2 of fold change and between CSH4 and WG350, CSH4 - CSH4 (S): log_2 of fold change between CSH4 and CSH4 salt, WG350 - WG350 (S): log_2 of fold change between WG350 and WG350 salt, CSH4 (S)- WG350 (S):): log_2 of fold change between CSH4 salt and WG350 salt

Supplementary Table 3: ODEs used for the kinetic simulations. (Related to figure 4)

Numerical simulation was set up with a fixed concentration of mRNA/ribosome complex (S). These complexes could either form nascent polypeptides (U) with a rate ktrans that is a collective rate constant for transcription/translation, or be inhibited with a rate constant kblock in the presence of a translation inhibitor (I). The pool of U could either degrade with a rate kdeg or fold with a rate constant of kf and reach the native state (F). We finally monitor the total amount of undegraded protein (U+F) after blocking translation after 300 s of starting the simulation (simulation start mimics induction). The reaction volume was kept at 1 liter.

ODEs:

d(U)/dt = -ReactionFlux1 - ReactionFlux2 + ReactionFlux3

d(F)/dt = ReactionFlux1

d(S)/dt = -ReactionFlux3 - ReactionFlux4

d(B)/dt = ReactionFlux4

d(I)/dt = -ReactionFlux4

Fluxes:

ReactionFlux1 = kf*U

ReactionFlux2 = kd*U

ReactionFlux3 = ktrans*S

ReactionFlux4 = ki*S*I

Parameter Values:

kf = 0.02 (s-1)

kdeg = 0.01 (s-1)

ktrans = 0.001(s-1)

ki = 0.0001 (M-1s-1)

cell volume = 1 (liters)

Initial Conditions (micromoles):

U = 0

F = 0

S= 1000

B = 0

I = 0

Dosing:

Delay: 300 s

1 mM of I added to reaction

Supplementary Table 4: Concentration of metabolites added to LB to measure effect of metabolites on Fluorescence *in vivo* (Related to Figure 7)

S. no.	Metabolite	Concentration (mM)
1	Alanine	200
2	Arginine	2.5
3	Betaine	200
4	Proline	200
5	Serine	200
6	TMAO	200
7	Aspartate	200
8	Malate	100
9	Malonate	100
10	Fumarate	100
11	Succinate	100
12	KCL	350
13	Sucrose	400
14	Sorbitol	400

Supplementary Table 5: Identity of genes along with the type of mutation observed in the evolved osmotolerant strains E5 and H8 on genome sequencing (Related to Figure 8)

Strain	Gene	Type
H8	mngA	Ins del
H8	hyfB	Ins del
H8	fucI	Ins del
H8	kefC	missense
H8	sgrR	missense
H8	mraY	missense
H8	ftsW	missense
H8	dxs	missense
H8	cstA	stop

H8	mngA	missense
H8	clpA	missense
H8	flgL	missense
H8	oppB	missense
H8	uidR	missense
H8	otsA	missense
E5	mngA	Ins del
E5	csiR	Ins del
E5	fucI	Ins del
E5	rihC	missense
E5	lptD	missense
E5	mraY	missense
E5	malZ	stop
E5	cyoA	missense
E5	paaH	missense
E5	sdaA	missense
