# Supplementary material for: Distinct metabolic states of a cell guide alternate fates of mutational buffering through altered proteostasis
Source: Nat Commun. 2020 Jun 10;11:2926. doi: 10.1038/s41467-020-16804-6 (PMC7286901; doi:10.1038/s41467-020-16804-6)
Supplement: Supplementary file 2 — Peer Review File [file 41467_2020_16804_MOESM2_ESM.docx]

Peer Review Information

**Journal:** Nature Communications

**Manuscript title:** Distinct metabolic states of a cell guide alternate fates of mutational buffering through altered proteostasis

**Corresponding author name(s):** Dr Chakraborty

**Editorial notes:**

**Transferred manuscripts**

This manuscript has been previously reviewed at another journal that is not operating a transparent peer review scheme. This document only contains reviewer comments and rebuttal letters for versions considered at *Nature Communications*.

**Transferred manuscripts (no peer review at *Nature Communications*)**

This manuscript has been previously reviewed at another journal that is not operating a transparent peer review scheme. The manuscript was considered suitable for publication without further review at *Nature Communications*.

**Redactions – transferred manuscripts (mention of previous referee reports from elsewhere)**

This manuscript has been previously reviewed at another journal that is not operating a transparent peer review scheme. This document only contains reviewer comments and rebuttal letters for versions considered at *Nature Communications*. Mentions of prior referee reports have been redacted.

**Redactions – transferred manuscripts (mention of the other journal)**

This manuscript has been previously reviewed at another journal that is not operating a transparent peer review scheme. This document only contains reviewer comments and rebuttal letters for versions considered at *Nature Communications*. Mentions of the other journal have been redacted.

**Redactions – unpublished data**

Parts of this Peer Review File have been redacted as indicated to maintain the confidentiality of unpublished data.

**Redactions – confidential patient information**

Parts of this Peer Review File have been redacted as indicated to maintain patient confidentiality.

**Redactions – published data**

Parts of this Peer Review File have been redacted as indicated to remove third-party material where no permission to publish could be obtained.

**Amendments – published data**

[Figure X/The figure on page X] in this Peer Review File has been amended to remove third-party material where no permission to publish could be obtained.

**Published data (credit line for image)**

[Figure X/The figure on page X] in this Peer Review File is reproduced with permission from [Copyright Holder].

**Redactions – reviewer opt-out**

Parts of this Peer Review File have been redacted as indicated as we could not obtain permission to publish the reports of reviewer ###.

**Reviewer comments in marked-up manuscript**

In their review of the [first/second/third/...] version of this manuscript, reviewer ### added their comments to the manuscript file. These comments, excluding minor textual revisions, have been copied into this Peer Review File.

**Reviewer comments & decisions:**

**Reviewer comments, first version:**

Reviewers' comments:

Reviewer #1 (Remarks to the Author):

This paper hypothesizes that changes in metabolic state (specifically osmolytes) plays a role on mutational buffering and therefore proteostasis more broadly. The authors posit that mutational buffering is specific to the protein readout, organismal context, and metabolic context. To address this question, the main study focuses on two e. coli strains and two protein. They perform several experiments to understand the specificity of why certain mutations are buffered in some genetic backgrounds but not others. They went very deep into the underlying reasons and do not come up with a silver bullet: the metabolomics and the transcriptomics do not reveal simple answers. Instead, the key experiments ground the results in more classic ideas of protein stability in different buffers. There is a lot in this paper, for example the mini-directed evolution experiment in high salt, that all dance around these same mysterious underlying causes. I hope the resulting datasets can be used for a more predictive approach as they are broadened, but as the authors caution, generalizing the conclusions may be difficult as each mutant needs to be considered on a case-by-base basis.

Specific points/questions:
Better defining “mutational buffering” up front is needed. This term can be used very broadly and differently depending on context.
Addressing how chemical chaperones might have differing effects on different protein folding states may be useful in light of what we know about the protein composition (i.e. how introducing salt will affect exposed hydrophobic sites, how hydrophobic in general are the proteins they looked at, etc.)
Authors should address how their result that molecular chaperones do not affect folding of isolated mutants since that is relatively unexpected. Is this perhaps because the proteins studied were non-native to their e. coli strain? This is related to the idea of a stimulatory role for metabolites on chaperone action (a broader indirect effect) rather than a specific role for metabolites stabilizing certain mutants?
This study goes deep into two strains, esp. with the metabolomics and transciptomics, but chemical perturbations that have effects on osmolarity could also be used. The evolved strain in high salt somewhat addresses this, but have the authors speculated or done any experiments on the effect of more transient or acute challenges and mutational buffering using these tools and libraries? (We are obviously interested in this as it is making us rethink some of our own chemical perturbations in deep mutational scans of ubiquitin)
The initial mutant the authors found in the Gm-R library were both to glycine and both in the same sequence space, and it is unclear what the WT residues are. The claim the authors make that they are less active in the transporter-deleted metabolic state may be a stretch. It would be nice to see where on the protein structure these mutants are – biophysically they might have a simpler explanation for their decrease in protein activity that is independent of the metabolites and similarly for their enhanced activity in the same strain with the addition of salt (i.e. may be restoring the charge disrupted by a glycine rather than mutational buffering).
The implications section could be grounded in a broader context tying together several threads in a proteostasis context rather than the larger leap to aging and metabolism. Eg. ATP levels as a protein stability/oligomerization agent: https://www.ncbi.nlm.nih.gov/pubmed/28522535, other classic literature on metabolic products and stability: https://www.ncbi.nlm.nih.gov/pmc/articles/PMC3996911/ , and amino acids as a refolding agent: https://www.ncbi.nlm.nih.gov/pubmed/15458311

We review non-anonymously, Erin Thompson and James Fraser, UCSF, and have posted our comments on the BioRxiv preprint as well.


Reviewer #3 (Remarks to the Author):

The manuscript entitled ‘Distinct metabolic states of a cell guide alternate fates of mutational buffering through altered proteostasis’ by Verma et al. aims to address the possibility of metabolic changes having an impact on cellular proteostasis. It is a very interesting new question in cell biology, which has so far gained only limited attention. As a context in which cellular proteostasis plays an important role, the authors picked mutation buffering in order to address the possible role of metabolite composition in mutation buffering.

I have carefully read the manuscript, and concluded that, although the performed experiments are designed and performed well, the work is missing several key experiments to be able to support the conclusions made here. In addition, even with the all the proper experiments, I have concerns about the novelty of the work:
1) Several groups have already proposed/demonstrated that metabolic changes are a part of cellular proteostasis (for example Morimoto, Dillin, and Krisko groups).
2) The senior author’s own work has already shown the involvement of TMAO osmolyte in mutation buffering (Nat Chem Biol. 2012; 8: 238–245.).

The conclusion that metabolites are members of the proteostasis network would be valid if it was shown that the osmolytes are contributing to already existing action of chaperones in buffering and that there is an actual crosstalk between chaperones and osmolyte synthesis. However, this study fails to do so.

Several specific comments:

1) First paragraph of the Results: the authors state that the two model proteins used in this study have different folds, which enables them to provide a fold-independent generality to their observations. This cannot be stated with having only two folds in total. To make such a statement, many more folds would have to be studied. I suggest to omit that statement from the manuscript.
2) Same paragraph: is it really important for in vivo mutation buffering and all the evolutionary implications that GFP is amenable to in vitro folding studies? That osmolytes are able to help with in vitro folding is already a well-known fact, and buffering is more of a systemic phenomenon.
3) Results, line 157-159: the authors state that the ability to take up metabolites from the medium affects metabolic networks and thus mutation buffering. This is at this point purely phenomenological and a huge possibility exists that there are confounding effects there that the authors haven’t taken into account. So we know that whatever is actually helping with buffering mutations in those conditions is triggered by adding some extra things in the medium; but what exactly is doing the job in the end, we don’t know.
4) Degradation experiments: a control with lon protease deletion should be performed to see if they will accumulate in the same manner or if there is something else going on there, like the different mutants being synthesized with different rates and therefore resulting in changes in fluorescence.
5) Results, line 247: mimicking a global increase in chaperone level by inducing heat shock and oxidative stress is not the way to go. So many things change under heat and oxidative stress, including the metabolic activity in cells. Why not simply overexpress chaperones, together with appropriate control, which would be overexpressing an inactivated version of each chaperone.
6) Results, line 252 on: these results raise a huge question if GFP is even a client for GroEL/ES and DnaK machinery. Without confirmation that it is, the whole study is not valid, and the results presented here leave a lot of doubt about that. If it is not a client, then the whole thing is just an effect of naturally produced osmolytes on GFP folding, something that is not at all novel, and that the authors themselves have shown before.
7) Results, line 305-306: the authors state that the results presented in the paragraph preceding those lines unravel the connection between mutation buffering and cellular metabolic status. However, they do not. Those results show that exogenous addition of many metabolites to growth media led to enhanced folding of GFP mutants. It’s nice, but that’s all. No good conclusion can be made about the cooperation between chaperones, buffering and metabolism there. I don’t see a need to even put those results there without further describing what they actually mean.
8) The in vitro evolution experiment: it shows that when put under osmotic stress, the bacteria will need increased osmolyte synthesis to adapt/survive and they will be able to use it to buffer mutations in the mutant GFP. The result is nice, but it goes along the same lines as other experiments. An interesting experiment would be to let a mutator E.coli evolve for generations (something like the Lenski experiment) and see if any of the generations would actually upregulate the osmolyte synthesis as a mutation buffering and/or adaptation strategy.
9) A key experiment would be the following: a mutator strain (to monitor the effects of natural mutations and not via reporter proteins) with and without GroEL/ES overexpressed – will the metabolic changes be triggered in those conditions? If yes, it would be essential to ‘uncouple’ the proteostasis from such metabolic changes and measure fitness in order to check if there will be any buffering.

Like this, without the actual effects of chaperones on the GFP folding, it seems we are looking at the chemical chaperoning alone and that is (i) not novel, and (ii) not about the metabolites being a part of the proteostasis network, but about them being able to act on their own.

**Author rebuttal, first version:**

Reviewers' comments:

Reviewer #1 (Remarks to the Author):

This paper hypothesizes that changes in metabolic state (specifically osmolytes) plays a role on mutational buffering and therefore proteostasis more broadly. The authors posit that mutational buffering is specific to the protein readout, organismal context, and metabolic context. To address this question, the main study focuses on two e. coli strains and two protein. They perform several experiments to understand the specificity of why certain mutations are buffered in some genetic backgrounds but not others. They went very deep into the underlying reasons and do not come up with a silver bullet: the metabolomics and the transcriptomics do not reveal simple answers. Instead, the key experiments ground the results in more classic ideas of protein stability in different buffers. There is a lot in this paper, for example the mini-directed evolution experiment in high salt, that all dance around these same mysterious underlying causes. I hope the resulting datasets can be used for a more predictive approach as they are broadened, but as the authors caution, generalizing the conclusions may be difficult as each mutant needs to be considered on a case-by-case basis.

This is a report to suggest that changes in physiological concentration of metabolites also change cellular proteostasis. We show that there is an alteration of proteostasis upon changing metabolism that is independent of the canonical chaperones. Thus, the focus of the work was to test if metabolites can alter proteostasis in vivo and change mutational buffering.

The reviewers have made an important point here - can we predict what mutations (in which proteins) will be buffered if we change the metabolism in a certain way?

We would require a more exhaustive dataset with multiple protein mutant libraries and with many different metabolic changes (that are quantified along with transcriptome) to develop a dataset that can be used for training machine learning models to predict the effect of metabolites in a mutant specific manner. We have already reported this complication exhaustively in (Dandage et al., 2015) and later in (Dandage et al., 2018). The small molecules have a complex effect depending on the protein fold and the mutations. Collecting this sort of dataset is complicated and involves substantial effort. The work is ongoing and the current one is first in this series where we start generating the datasets required for this type of analysis.

Specific points/questions:
1. Better defining “mutational buffering” up front is needed. This term can be used very broadly and differently depending on context.

We have tried to define this in the introduction and **Figure 1**. We have also streamlined the introduction to make this concept clearer.

2. Addressing how chemical chaperones might have differing effects on different protein folding states may be useful in light of what we know about the protein composition (i.e. how introducing salt will affect exposed hydrophobic sites, how hydrophobic in general are the proteins they looked at, etc.)

Suggestion duly taken and now we have added a paragraph in the implications section highlighting the same. Additionally, we have added structural analysis of the mutants (please see a response below) to indicate the type of mutations that seem to be buffered by altered metabolite composition.

3. Authors should address how their result that molecular chaperones do not affect folding of isolated mutants since that is relatively unexpected. Is this perhaps because the proteins studied were non-native to their e. coli strain?

While there are recent reports showing that not all mutations are dependent upon chaperones for folding(Tripathi et al., 2016), it is a common knowledge that overexpression of specific chaperones do not help folding of all proteins (or mutants).

We have now included a section showing how the GFP model is able to detect alteration in canonical proteostasis machinery (line 300-310, **Figure 5H, S4E**). There are two important points we would like to clarify. First, from the library of mutants, few were isolated that showed different fluorescence (folding) in two different strains. These mutants were not chaperone dependent, although the library had other mutants that were chaperone dependent. Second, not all mutants are chaperone dependent. We still do not understand what types of mutations (in a protein specific manner) leads to dependence on which chaperone. We have isolated GroEL/ES dependent GFP mutants in a collaborative study using the same mutant library. The unpublished manuscript is attached with the answers for reference.

4. This is related to the idea of a stimulatory role for metabolites on chaperone action (a broader indirect effect) rather than a specific role for metabolites stabilizing certain mutants?

This is one possibility however; the other possibility is that metabolites (like osmolytes) alter solvent structure or bind to (un)structured regions to help folding. In case metabolites only potentiated the function of molecular chaperones we would have seen chaperone dependent mutants to be hyperactive in presence of these metabolites (upon altering metabolism - WG350(S)). This is not the case (the mutants are not found to be dependent on chaperones), the chaperone-dependent mutants are not altered, but mutants that are altered are inherently dependent on the small molecules. We believe this is indeed due to the ability of the small molecules to act on the proteins (solvent) themselves.

It is unexpected because there have been no reports of metabolites being a part of the proteostasis network and its capacity to function independently. As explained in the introduction taking an endogenous protein as a sensor would restrict our capacity to uncouple effect on protein folding from other compensatory effects. However, not all mutants in the two libraries used in the study are independent of molecular chaperones (sGFP from the same library is a substrate for GroEL). We do not find stimulatory role of metabolite on molecular chaperones in buffering of these mutants as these mutants are not assisted by GroEL/ES or dnaK/J/GrpE machinery both *in vivo* and *in vitro* as shown in **Figure 5D, 5F**.

5.This study goes deep into two strains, esp. with the metabolomics and transcriptomics, but chemical perturbations that have effects on osmolarity could also be used.

A. We agree and this is what initiated us to use NaCl in the later part of the work. However, as the starting point we did not want to alter osmolarity directly or abruptly as it has been shown earlier that changes in osmolarity leads to alteration in a large number of proteostasis pathways including translation, folding and degradation. In a separate section of the manuscript (**Figure 7**) we have used chemical perturbations that cause large osmolarity changes (400mM Sucrose, 400mm Sorbitol to CSH4 strain) and did not observe buffering. This suggested that osmotic shock alone is not altering the buffering in CSH4 but the cellular response to osmotic shock (the final cellular milieu) determined folding capacity of these cells when probed with the mutants we isolated.
B. We did use NaCl from outside in the strains chosen but the concentration was so chosen that it did not affect the growth of the organisms significantly. Transcriptomics and metabolomics of both the strains under physiological conditions of growth and with mild osmotic shock were recorded and analyzed (This section is now expanded as suggested). We made sure using these that the canonical pathways of proteostasis are not significantly altered. This assured that we could distinguish the effect of metabolites and chaperones on folding *in vivo.*
C. We did not use other chemicals that induce a strong osmotic shock as this would convolute the analysis of the mutants that are buffered. It would be difficult to resolve if the molecules used for the treatment (present at non-physiological concentrations) led to buffering (this was done in our previous work (Bandyopadhyay et al., 2012)), or if the cellular response to the osmotic shock led to the difference in folding observed.
D. Instead, we used an array of small molecules at moderately high concentrations to modify the cellular metabolism (**Figure 7**) and check if these molecules can affect folding.

6. The evolved strain in high salt somewhat addresses this, but have the authors speculated or done any experiments on the effect of more transient or acute challenges and mutational buffering using these tools and libraries? (We are obviously interested in this as it is making us rethink some of our own chemical perturbations in deep mutational scans of ubiquitin)

We have done some preliminary experiments with acute stress (data shown below) which is similar to what we obtained with chronic stress. However, with the current reporter (mutant GFP/Wt mCherry) it is not possible to follow an acute response in real time for the different strains. We are also interested in understanding the difference between acute and chronic alterations in metabolism (or stress response), the development of a suitable tool to study this however is outside the scope of this current work.

**Figure:** Plot for gel-based quantification of intact GFP (left) and representative blots (right) to chase degradation of C1 GFP in presence and absence of acute osmotic stress (15min) to CSH4 and WG350 followed by protein induction for 5 min and translation arrest by Chloramphenicol. GFP intact is shown by immunoblotting with anti-GFP antibody.

7. The initial mutant the authors found in the Gm-R library were both to glycine and both in the same sequence space, and it is unclear what the WT residues are.

We have now included the original residues in the naming convention. The reference has been provided for Gm-R in the results however, we will include the complete sequence in supplementary or bio project if required. The mutant library used were glycine-duplet substitution library as shown schematically in **Figure S1A**

8. The claim the authors make that they are less active in the transporter-deleted metabolic state may be a stretch. It would be nice to see where on the protein structure these mutants are – biophysically they might have a simpler explanation for their decrease in protein activity that is independent of the metabolites and similarly for their enhanced activity in the same strain with the addition of salt (i.e. may be restoring the charge disrupted by a glycine rather than mutational buffering).

If a mutant protein is less active than WT, it may have nothing to with proteostasis but if they are differently active in two different strains, it may be either due to difference in protein amount (proteostasis) or due to difference in specific activity. With GFP we confirm that these mutants do not have differences in specific activity. We demonstrate that this is due to different steady state levels of mutant proteins in the different strains by using GFP (*in vivo* quantitation/biophysics). We show that it is dependent on cellular proteostasis by *in vivo* degradation experiments. Additionally, we provide evidence that these mutants are most probably destabilized mutants and not inactive ones with detailed structural analysis (now added in **Figure 2F, 2H (Gm-R) and 3H, 3I (GFP)**). This is also supported by the *in vitro* refolding experiments in presence and absence of chemical chaperones like TMAO that aid folding of some of these mutants. We had not included a comprehensive structural analysis of the mutants as we had published it earlier(Bandyopadhyay et al., 2012; Dandage et al., 2015). We have now included some analysis of the mutants to support our claims related to protein folding. Most of these mutants are predicted to affect protein stability, while majority do not lead to any charge alterations ruling out simple effect of salt on stability. We have also shown that NaCl directly does not increase folding of the mutants *in vitro*. The mutants GG1(I26G) and GG2(I27G, R28G) of Gm-R shows difference in activity between CSH4 and WG350 in absence of salt. So, the difference in their activity is not salt-dependent *in vivo*.

9. The implications section could be grounded in a broader context tying together several threads in a proteostasis context rather than the larger leap to aging and metabolism. Eg. ATP levels as a protein stability/oligomerization agent: <https://www.ncbi.nlm.nih.gov/pubmed/28522535>, other classic literature on metabolic products and stability: <https://www.ncbi.nlm.nih.gov/pmc/articles/PMC3996911/> , and amino acids as a refolding agent: <https://www.ncbi.nlm.nih.gov/pubmed/15458311>

The implication section is now rewritten based on the suggestion.

We review non-anonymously, Erin Thompson and James Fraser, UCSF, and have posted our comments on the BioRxiv preprint as well.

We thank the reviewer for his help in understanding certain questions through BiorXiv.

Reviewer #3 (Remarks to the Author):

The manuscript entitled ‘Distinct metabolic states of a cell guide alternate fates of mutational buffering through altered proteostasis’ by Verma et al. aims to address the possibility of metabolic changes having an impact on cellular proteostasis. It is a very interesting new question in cell biology, which has so far gained only limited attention. As a context in which cellular proteostasis plays an important role, the authors picked mutation buffering in order to address the possible role of metabolite composition in mutation buffering.

I have carefully read the manuscript, and concluded that, although the performed experiments are designed and performed well, the work is missing several key experiments to be able to support the conclusions made here. In addition, even with the all the proper experiments, I have concerns about the novelty of the work:

We thank the reviewer for his critical comments which led us to rewrite and rework large sections of the manuscript to highlight the contributions made by this report. Namely 1) Showing that metabolites are integral to proteostasis (please see response to this reviewer below 10), and 2) Changes in metabolite pool can alter mutational buffering by changing proteostasis.

1) Several groups have already proposed/demonstrated that metabolic changes are a part of cellular proteostasis (for example Morimoto, Dillin, and Krisko groups).

We agree that several reports have looked into the link of proteostasis and metabolism. The groups mentioned have contributed significantly in understanding how translation, autophagy and even chaperone levels are linked to metabolic signals (through TOR pathway(Perić et al., 2017)). As we had stated in the introduction (lines 33-37 & 69 to 77), our aim was to understand if the metabolites themselves (partially independent of chaperones) can also perturb cellular proteostasis. This is the reason we had chosen *E. coli* as the model system. How metabolic networks part form part of larger transcriptional/translational network to regulate the canonical components (molecular chaperones, translation, degradation) was not the focus of this work although this forms an essential part of the question that is being addressed carefully by other groups(Perić et al., 2017; Tabrez, Sharma, Jain, Siddiqui, & Mukhopadhyay, 2017)

There has been no report of metabolite changes regulating proteostasis capacity till date.

2) The senior author’s own work has already shown the involvement of TMAO osmolyte in mutation buffering (Nat Chem Biol. 2012; 8: 238–245.).

We thank the reviewer for pointing this out. We have clarified the confusion in the introduction section (Lines 71 to 77). Our earlier work was performed with excess of chemical chaperones added to growth medium. This did not however show that endogenous metabolites at their physiological concentration contribute to protein folding in the cell. This study reveals the contribution of metabolites at their **endogenous concentrations** in regulating cellular proteostasis.

The conclusion that metabolites are members of the proteostasis network would be valid if it was shown that the osmolytes are contributing to already existing action of chaperones in buffering and that there is an actual crosstalk between chaperones and osmolyte synthesis. However, this study fails to do so.

We agree that this is one interesting aspect that needs further investigation and this is one of the questions we are currently investigating. However, in this current work we are trying to implicate that metabolites can regulate protein folding even independently (this is one of the novelties of the work). Their cross-talk with chaperone machinery had been investigated *in vitro* by the Goloubinoff group (Diamant, Eliahu, Rosenthal, & Goloubinoff, 2001), and we agree that it will be important to follow up these results *in vivo*. For this we would require chaperone-dependent mutants to be buffered in different metabolic states (Please see response 4 in the answers to the previous reviewer).

Several specific comments:

1) First paragraph of the Results: the authors state that the two model proteins used in this study have different folds, which enables them to provide a fold-independent generality to their observations. This cannot be stated with having only two folds in total. To make such a statement, many more folds would have to be studied. I suggest to omit that statement from the manuscript.

We agree with this comment. The suggestion is duly taken and is omitted in the revised version. We only wished to state the following - "that the buffering effect is seen in two very diverse protein folds we can conclude that the effect is not restricted to the specific fold of one protein".

2) Same paragraph: is it really important for in vivo mutation buffering and all the evolutionary implications that GFP is amenable to in vitro folding studies? That osmolytes are able to help with in vitro folding is already a well-known fact, and buffering is more of a systemic phenomenon.

No. there is a miscommunication here. That GFP is amenable to *in vitro* refolding is technically important for us to reconstitute the chaperone dependence of the isolated mutants *in vitro*. To see if the buffered mutants actually have a folding defect and is assisted by some molecules that are different between strains or conditions where we see differences in fluorescence. Actually we had shown earlier that Wt GFP is not dependent on a lot of chemical chaperones for folding (Dandage et al., 2018). Even here we find that different mutants have difference in their dependence on chemical chaperones. So, although it is well known that some small molecules can act as chemical chaperones, their substrate specificity is not known.

Regarding the concept of GFP folding and the systemic effect of buffering: here we are concerned solely with genetic buffering of non-synonymous mutations (now clarified in Line 68 to 70). These mutations may cause the protein to misfold and hence be degraded. If concentrations of molecules that assist the protein change *in vivo*, we will be able to observe difference in the amount of folded protein as a function of the assisting molecule. This effect is to some extent known for molecular chaperones(Bandyopadhyay et al., 2012; Tokuriki, Stricher, Serrano, & Tawfik, 2008) , however this is not known for metabolites (other than the effect of ATP or polyphosphates on protein aggregation and in specific stresses).

3) Results, line 157-159: the authors state that the ability to take up metabolites from the medium affects metabolic networks and thus mutation buffering. This is at this point purely phenomenological and a huge possibility exists that there are confounding effects there that the authors haven’t taken into account. So, we know that whatever is actually helping with buffering mutations in those conditions is triggered by adding some extra things in the medium; but what exactly is doing the job in the end, we don’t know.

This experiment only supplements the data that we have obtained *in vitro*. Molecules that help *in vitro* shows some effect *in vivo* but only in certain strains. We therefore are aware of the complications and the importance of indirect effects. However, we do know that these are protein folding effects as we have proper controls (folded proteins that are independent of chaperones, mCherry in the same construct as well as Wt GFP in parallel experiments). Addition of excess metabolites are known to change metabolism and we use this just to highlight the point that metabolic alterations caused by adding external metabolites also change protein folding for these mutants (while these mutants are not really dependent on the most abundant molecular chaperones).

It is true that we don’t know what exactly is doing the job but it is certainly not molecular chaperones rather, the components of complex cellular chemical milieu containing metabolites. Which is not phenomenological as differences are captured in metabolite levels and are partially reconstituted ***in vitro*** using pure small molecules.

4) Degradation experiments: a control with lon protease deletion should be performed to see if they will accumulate in the same manner or if there is something else going on there, like the different mutants being synthesized with different rates and therefore resulting in changes in fluorescence.

We think that this indeed added to the quality of control experiments. We have done these experiments as suggested and have incorporated them in **Figure 5I, S4F**. This data clearly shows that these mutants are recognized by the DnaK system as compromised folders and are degraded through the Lon proteasome system.

5) Results, line 247: mimicking a global increase in chaperone level by inducing heat shock and oxidative stress is not the way to go. So many things change under heat and oxidative stress, including the metabolic activity in cells. Why not simply overexpress chaperones, together with appropriate control, which would be overexpressing an inactivated version of each chaperone.

There seems to have been a miscommunication here. We agree completely with the reviewer that heat shock and any other stress is not a specific way of increasing chaperone levels. We therefore had already presented that overexpression of specific chaperone systems do not lead to better folding of these mutants *in vivo* (**Figure 5D**)

6) Results, line 252 on: these results raise a huge question if GFP is even a client for GroEL/ES and DnaK machinery. Without confirmation that it is, the whole study is not valid, and the results presented here leave a lot of doubt about that.

We think it is an important point raised by both the reviewers and we now have added extensive data (**please see response no. 3 to reviewer #1 for details**) to support that GFP mutant library indeed has many mutants that are dependent on the endogenous chaperones.

We here have shown mutants buffered by metabolites but as a control we have also shown unchanged degradation rates of the mutant sGFP not buffered by metabolites. This mutant is a substrate of GroEL/ES machinery (Sadat et. al. manuscript attached).  there are other mutants in the library screen of both GFP and GmR which are substrates of molecular chaperones

If it is not a client, then the whole thing is just an effect of naturally produced osmolytes on GFP folding, something that is not at all novel, and that the authors themselves have shown before.

We think that the reviewer believes that naturally produced osmolytes are already well accepted to help protein folding *in vivo* while the whole literature is full of data on the contrary even for as well characterized molecules like Trehalose(Singer & Lindquist, 1998). This is primarily because the previous studies lacked the ability to check protein folding using mutant libraries. Here we show that metabolite pools indeed change folding inside cells. So, the concept of osmolytes having a role in folding in vivo and they regulating protein folding in a mutant specific manner is not well accepted. Again, we would like to reiterate that we had shown the effect of externally added non-physiological levels of chemical chaperones. Here we show that endogenously synthesized metabolites may also contribute to intracellular protein folding.

7) Results, line 305-306: the authors state that the results presented in the paragraph preceding those lines unravel the connection between mutation buffering and cellular metabolic status. However, they do not. Those results show that exogenous addition of many metabolites to growth media led to enhanced folding of GFP mutants. It’s nice, but that’s all. No good conclusion can be made about the cooperation between chaperones, buffering and metabolism there. I don’t see a need to even put those results there without further describing what they actually mean.

We think that the reviewer believes that for metabolites to act as genetic buffers they should work through the chaperones. While this is an interesting model, we claim that metabolites also have independent effects. Here we added different amino acids for example to change cellular metabolism. We also see that the folding capacity of the cells change (in a strain specific manner). We believe that the only conclusion is that metabolic alterations lead to change in activity of mutant proteins that have some specific folding defects. This is the only conclusion and we use this to supplement the whole body of work (changing metabolism by genetic means and by osmotic shock). We would be happy to remove this whole figure if the reviewer thinks appropriate but have left it as such to point out that chemical perturbation of metabolism of also changes mutational buffering in *E. coli*.

**Figure 6A** shows how same concentration of different metabolites when added to the refolding buffer *in vitro* accelerates the refolding rates of individual GFP mutants differently on the other hand data from **Figure 7** clearly shows that altering cellular metabolism by supplementing different metabolite in excess to the LB medium instead of osmotic stress by NaCl modifies the cellular buffering potential differently resulting in metabolite-mutant pair specific fluorescence phenotype. Taken together, these unravel the connection between mutation buffering and cellular metabolic status. Further, as shown in **Figure 6B**, protein free lysate of the cell (independent of any canonical chaperoning machinery) independently assist in refolding of the mutants substantially. All highlighting the contribution of metabolites in mutational buffering both *in vivo* and *in vitro.* **Note: We do not claim cooperation between chaperones, buffering and metabolism here**8) The in vitro evolution experiment: it shows that when put under osmotic stress, the bacteria will need increased osmolyte synthesis to adapt/survive and they will be able to use it to buffer mutations in the mutant GFP. The result is nice, but it goes along the same lines as other experiments.

We agree that this is exactly what we were trying to point out to. Accumulation of intracellular metabolites (and not only the canonical osmolytes) can change cellular protein folding. Interestingly there is no evidence in literature supporting this. It is important to note that we were performing all the buffering experiments in the absence of osmotic shock. This was to rule out that alteration of folding was due to changes in extracellular osmolarity.

That is why it is a supporting evidence nailing the point that metabolic alterations are the way of mutational buffering through maintaining proteostasis. We do not understand reviewer's concern here!

An interesting experiment would be to let a mutator E.coli evolve for generations (something like the Lenski experiment) and see if any of the generations would actually upregulate the osmolyte synthesis as a mutation buffering and/or adaptation strategy.

This is an exciting suggestion. We have evolved more than 40 thermotolerant strains work a separate ongoing work and we do have mutators in them. These strains, however, do not buffer the mutations buffered by evolved osmotolerant or strains having altered metabolite pool due to osmotic homeostasis as in case of WG350 (**as shown below**). This is likely due to difference is osmotic composition of the strains evolving under a different selection pressure. This will be followed up as suggested. However, inclusion of this will be outside the direct scope of the manuscript as more careful analysis of these strains would need to be performed to obtain conclusive results regarding buffering (genomics/transcriptomics/metabolomics/mutant-GFP isolation/characterization). Connecting the changes in metabolome of these strains to buffering will require non-trivial analysis as discussed in the next response.

**Figure:** Histogram for fluorescence of GFP mutants C1 and C6 represented as GFP/mCherry fluorescence, in GFP channel and mCherry channel in unevolved strain (Δ*proA*) and evolved thermotolerant strains (Tt C4 and Tt D10).

9) A key experiment would be the following: a mutator strain (to monitor the effects of natural mutations and not via reporter proteins) with and without GroEL/ES overexpressed – will the metabolic changes be triggered in those conditions? If yes, it would be essential to ‘uncouple’ the proteostasis from such metabolic changes and measure fitness in order to check if there will be any buffering.

We agree that mutator strains may change metabolism in order to sustain proteostasis. The disadvantage of using a mutator strain will be the mutations on its endogenous proteins altering several pathways eliciting several buffering mechanisms making it difficult to address proteostasis specifically. Additionally, as we have shown in a collaborative study (preprint attached) GroEL/ES is not a general buffer of protein folding mutants and only buffers mutations that it can handle. Thus overexpression of GroEL/ES will not be able to supplement defects of many proteins and hence there will be changes in the cell which are independent of proteostasis(Wyganowski, Kaltenbach, & Tokuriki, 2013).

10) Like this, without the actual effects of chaperones on the GFP folding, it seems we are looking at the chemical chaperoning alone and that is (i) not novel, and (ii) not about the metabolites being a part of the proteostasis network, but about them being able to act on their own.

Without the actual effect of chaperones, we are indeed looking at the under-rated potential of the metabolites in helping folding. The confusion seems to be between chemical chaperoning (shown so far either in vitro or by exogenous supplementation) and mutation buffering by metabolites (not just previously characterized chemical chaperones) at their endogenous concentrations *in vivo*. This is the maiden study that reports the essentiality of cellular metabolism in mutational buffering though its effect on proteostasis. We here report that metabolites can work independently - this is an important discovery - and maybe in collaboration with chaperones to maintain proteostasis. If this was known the literature would have been updated placing metabolites along with molecular chaperones in the proteostasis network, which is not the case!

The reviewer in his last sentence **“but about them being to act on their own”** has indeed highlighted the importance of this work. Proteostasis is defined as mechanisms that maintain the homeostasis of protein biogenesis. If metabolites are indeed affecting the biogenesis of certain proteins (or mutants) we believe that they should be considered as part of the proteostasis network even if they are not macromolecules.

To highlight this, we have now changed the introduction and the implication to highlight the gaps in knowledge and the importance of this finding in the context of *E. coli* and other prokaryotes.

**Reviewer comments, second version:**

Reviewer #1 (Remarks to the Author):

This paper hypothesizes that changes in metabolic state (specifically osmolytes) plays a role on mutational buffering and therefore proteostasis more broadly. The authors posit that mutational buffering is specific to the protein readout, organismal context, and metabolic context. To address this question, the main study focuses on two e. coli strains and two protein. They perform several experiments to understand the specificity of why certain mutations are buffered in some genetic backgrounds but not others. They went very deep into the underlying reasons and do not come up with a silver bullet: the metabolomics and the transcriptomics do not reveal simple answers. Instead, the key experiments ground the results in more classic ideas of protein stability in different buffers. There is a lot in this paper, for example the mini-directed evolution experiment in high salt, that all dance around these same mysterious underlying causes. I hope the resulting datasets can be used for a more predictive approach as they are broadened, but as the authors caution, generalizing the conclusions may be difficult as each mutant needs to be considered on a case-by-case basis.

This is a report to suggest that changes in physiological concentration of metabolites also change cellular proteostasis. We show that there is an alteration of proteostasis upon changing metabolism that is independent of the canonical chaperones. Thus, the focus of the work was to test if metabolites can alter proteostasis in vivo and change mutational buffering.

The reviewers have made an important point here - can we predict what mutations (in which proteins) will be buffered if we change the metabolism in a certain way?

We would require a more exhaustive dataset with multiple protein mutant libraries and with many different metabolic changes (that are quantified along with transcriptome) to develop a dataset that can be used for training machine learning models to predict the effect of metabolites in a mutant specific manner. We have already reported this complication exhaustively in (Dandage et al., 2015) and later in (Dandage et al., 2018). The small molecules have a complex effect depending on the protein fold and the mutations. Collecting this sort of dataset is complicated and involves substantial effort. The work is ongoing and the current one is first in this series where we start generating the datasets required for this type of analysis.

We agree, it is a LOT of work to make and test protein mutant libraries. If the authors do not wish to do the experiments to get a more exhaustive dataset with multiple protein mutant libraries, they should back off some of their strong claims that this is so generalizable and scale back their statements to be specific to the system & proteins they examined. Perhaps the discussion section would be a better place for the authors to discuss the broader implications of what mutational buffering at the level of these two proteins means.

Specific points/questions:

1. Better defining “mutational buffering” up front is needed. This term can be used very broadly

and differently depending on context.

We have tried to define this in the introduction and **Figure 1**. We have also streamlined the introduction to make this concept clearer.

Figure 1 makes this much more clear and is a valuable addition to the manuscript. note for

2. Addressing how chemical chaperones might have differing effects on different protein folding states may be useful in light of what we know about the protein composition (i.e. how

introducing salt will affect exposed hydrophobic sites, how hydrophobic in general are the proteins they looked at, etc.)

Suggestion duly taken and now we have added a paragraph in the implications section highlighting the same. Additionally, we have added structural analysis of the mutants (please see a response below) to indicate the type of mutations that seem to be buffered by altered metabolite composition.

The implications paragraph covers this point sufficiently.

3. Authors should address how their result that molecular chaperones do not affect folding of isolated mutants since that is relatively unexpected. Is this perhaps because the proteins studied were non-native to their e. coli strain?

While there are recent reports showing that not all mutations are dependent upon chaperones for folding(Tripathi et al., 2016), it is a common knowledge that overexpression of specific chaperones do not help folding of all proteins (or mutants).

We have now included a section showing how the GFP model is able to detect alteration in canonical proteostasis machinery (line 300-310, **Figure 5H, S4E**). There are two important points we would like to clarify. First, from the library of mutants, few were isolated that showed different fluorescence (folding) in two different strains. These mutants were not chaperone dependent, although the library had other mutants that were chaperone dependent. Second, not all mutants are chaperone dependent. We still do not understand what types of mutations (in a protein specific manner) leads to dependence on which chaperone. We have isolated GroEL/ES dependent GFP mutants in a collaborative study using the same mutant library. The

One reason we find the paper confusing is that they really are referring to [general] mutational buffering as two things: 1 - due to molecular chaperon assistance and 2 - through metabolite dependent changes on protein folding. Yet they include statements like this one in the abstract throughout the paper:

“

Since proteostasis can modulate mutational buffering, if change in metabolism has the ability to change proteostasis, arguably, it should also alter mutational buffering. Building on this, we find that altered cellular metabolic states in *E. coli* buffer distinct mutations. Buffered-mutants had folding problems *in vivo* and were differently chaperoned in different metabolic states

“

where they seem to be mixing the two mutational buffering mechanisms or leaning too heavily on the assistance from molecular chaperones to explain changes that seem to be due to metabolite dependent changes

Again, we think that the authors’ are trying to say something very specific about 1) proteostasis through molecular chaperones 2) mutational buffering through metabolites independently and 3) how metabolites/osmolytes affect chaperones. However, we do not in general feel that they

were very clear or specific in their language throughout the paper in clarifying what exactly they meant. For example, many times in the paper the authors use “proteostasis”, which we associate with molecular chaperones. However, it is clear from their language that they are using mutational buffering and proteostasis interchangeably at this point

4. This is related to the idea of a stimulatory role for metabolites on chaperone action (a broader indirect effect) rather than a specific role for metabolites stabilizing certain mutants?

This is one possibility however; the other possibility is that metabolites (like osmolytes) alter solvent structure or bind to (un)structured regions to help folding. In case metabolites only potentiated the function of molecular chaperones we would have seen chaperone dependent mutants to be hyperactive in presence of these metabolites (upon altering metabolism - WG350(S)). This is not the case (the mutants are not found to be dependent on chaperones), the chaperone-dependent mutants are not altered, but mutants that are altered are inherently dependent on the small molecules. We believe this is indeed due to the ability of the small molecules to act on the proteins (solvent) themselves.

It is unexpected because there have been no reports of metabolites being a part of the proteostasis network and its capacity to function independently. As explained in the introduction taking an endogenous protein as a sensor would restrict our capacity to uncouple effect on protein folding from other compensatory effects. However, not all mutants in the two libraries used in the study are independent of molecular chaperones (sGFP from the same library is a substrate for GroEL). We do not find stimulatory role of metabolite on molecular chaperones in buffering of these mutants as these mutants are not assisted by GroEL/ES or dnaK/J/GrpE machinery both *in vivo* and *in vitro* as shown in **Figure 5D, 5F**.

I think the points made in the first paragraph of this response are a good indication that it is not a broader indirect effect. We agree.

5.This study goes deep into two strains, esp. with the metabolomics and transcriptomics, but chemical perturbations that have effects on osmolarity could also be used.

A. We agree and this is what initiated us to use NaCl in the later part of the work. However, as the starting point we did not want to alter osmolarity directly or abruptly as it has been shown earlier that changes in osmolarity leads to alteration in a large number of proteostasis pathways including translation, folding and degradation. In a separate section of the manuscript (**Figure 7**) we have used chemical perturbations that cause large osmolarity changes (400mM Sucrose, 400mm Sorbitol to CSH4 strain) and did not observe buffering. This suggested that osmotic shock alone is not altering the buffering in CSH4 but the cellular response to osmotic shock (the final cellular milieu) determined folding capacity of these cells when probed with the mutants we isolated.
B. We did use NaCl from outside in the strains chosen but the concentration was so chosen that it did not affect the growth of the organisms significantly. Transcriptomics and metabolomics of both the strains under physiological conditions of growth and with mild osmotic shock were recorded and analyzed (This section is now expanded as suggested). We made sure using these that the canonical pathways of proteostasis are not significantly altered. This assured that we could distinguish the effect of metabolites and chaperones on folding *in vivo.*
C. We did not use other chemicals that induce a strong osmotic shock as this would convolute the analysis of the mutants that are buffered. It would be difficult to resolve if the molecules used for the treatment (present at non-physiological concentrations) led to buffering (this was done in our previous work (Bandyopadhyay et al., 2012)), or if the cellular response to the osmotic shock led to the difference in folding observed.
D. Instead, we used an array of small molecules at moderately high concentrations to modify the cellular metabolism (**Figure 7**) and check if these molecules can affect folding.

The authors’ response has sufficiently addressed comment #5. Figure 7 shows the results of the KCL, sucrose, and sorbitol supplementation and Figure S1B shows the growth results with NaCl addition.

6. The evolved strain in high salt somewhat addresses this, but have the authors speculated or done any experiments on the effect of more transient or acute challenges and mutational buffering using these tools and libraries? (We are obviously interested in this as it is making us rethink some of our own chemical perturbations in deep mutational scans of ubiquitin)

We have done some preliminary experiments with acute stress (data shown below) which is similar to what we obtained with chronic stress. However, with the current reporter (mutant GFP/Wt mCherry) it is not possible to follow an acute response in real time for the different strains. We are also interested in understanding the difference between acute and chronic alterations in metabolism (or stress response), the development of a suitable tool to study this however is outside the scope of this current work.

**Figure:** Plot for gel-based quantification of intact GFP (left) and representative blots (right) to chase degradation of C1 GFP in presence and absence of acute osmotic stress (15min) to CSH4 and WG350 followed by protein induction for 5 min and translation arrest by Chloramphenicol. GFP intact is shown by immunoblotting with anti-GFP antibody.

We agree it is outside the scope of the current work, and their response is fine. We think we were more curious on this point than demanding they address it in this paper.

7. The initial mutant the authors found in the Gm-R library were both to glycine and both in the same sequence space, and it is unclear what the WT residues are.

We have now included the original residues in the naming convention. The reference has been provided for Gm-R in the results however, we will include the complete sequence in supplementary or bio project if required. The mutant library used were glycine-duplet substitution library as shown schematically in **Figure S1A**

**I think the complete sequence would be a good addition to the supplement. Figure S1A makes the library more clear and is a good addition.**

8. The claim the authors make that they are less active in the transporter-deleted metabolic state may be a stretch. It would be nice to see where on the protein structure these mutants are – biophysically they might have a simpler explanation for their decrease in protein activity that is independent of the metabolites and similarly for their enhanced activity in the same strain with the addition of salt (i.e. may be restoring the charge disrupted by a glycine rather than

mutational buffering).

If a mutant protein is less active than WT, it may have nothing to with proteostasis but if they are differently active in two different strains, it may be either due to difference in protein amount (proteostasis) or due to difference in specific activity. With GFP we confirm that these mutants do not have differences in specific activity. We demonstrate that this is due to different steady state levels of mutant proteins in the different strains by using GFP (*in vivo* quantitation/biophysics). We show that it is dependent on cellular proteostasis by *in vivo* degradation experiments. Additionally, we provide evidence that these mutants are most probably destabilized mutants and not inactive ones with detailed structural analysis (now added in **Figure 2F, 2H (Gm-R) and 3H, 3I (GFP)**). This is also supported by the *in vitro* refolding experiments in presence and absence of chemical chaperones like TMAO that aid folding of some of these mutants. We had not included a comprehensive structural analysis of the mutants as we had published it earlier(Bandyopadhyay et al., 2012; Dandage et al., 2015). We have now included some analysis of the mutants to support our claims related to protein folding. Most of these mutants are predicted to affect protein stability, while majority do not lead to any charge alterations ruling out simple effect of salt on stability. We have also shown that NaCl directly does not increase folding of the mutants *in vitro*. The mutants GG1(I26G) and GG2(I27G, R28G) of Gm-R shows difference in activity between CSH4 and WG350 in absence of salt. So, the difference in their activity is not salt-dependent *in vivo*.

While we still think some structural analysis would help, the conservation plots in figures 2 & 3 start to tell us we were asking for in this comment.

9. The implications section could be grounded in a broader context tying together several threads in a proteostasis context rather than the larger leap to aging and metabolism. Eg. ATP levels as a protein stability/oligomerization

agent: [https://www.ncbi.nlm.nih.gov/pubmed/28522535](https://www.ncbi.nlm.nih.gov/pubmed/28522535,), other classic literature on metabolic products and stability: <https://www.ncbi.nlm.nih.gov/pmc/articles/PMC3996911/> , and amino acids as a refolding agent: <https://www.ncbi.nlm.nih.gov/pubmed/15458311>

The implication section is now rewritten based on the suggestion.

I think the re-written implication section is good.

We review non-anonymously, Erin Thompson and James Fraser, UCSF, and have posted our comments on the BioRxiv preprint as well.

We thank the reviewer for his help in understanding certain questions through BiorX

Reviewers' comments:

Reviewer #3 (Remarks to the Author):

I have now carefully read and analyzed the manuscript by Chakraborty and colleagues, as well as their rebuttal. I find that all my concerns have been addressed by the authors, at least the most important ones. I still believe that the crucial evidence on how important metabolome is in mutation buffering can arise only via an experiment such as with mutator E.coli, as discussed in my initial comments as well as the rebuttal letter (even though i personally strongly believe that it is). However, this is an editorial decision, and i would like to congratulate the authors on the interesting results presented here.

**Author rebuttal, second version:**

Reviewer #1 (Remarks to the Author):

This paper hypothesizes that changes in metabolic state (specifically osmolytes) plays a role onmutational buffering and therefore proteostasis more broadly. The authors posit that mutational buffering is specific to the protein readout, organismal context, and metabolic context. To address this question, the main study focuses on two e. coli strains and two protein. They perform several experiments to understand the specificity of why certain mutations are buffered in some genetic backgrounds but not others. They went very deep into the underlying reasons and do not come up with a silver bullet: the metabolomics and the transcriptomics do not reveal simple answers. Instead, the key experiments ground the results in more classic ideas of protein stability in different buffers. There is a lot in this paper, for example the mini-directed evolution experiment in high salt, that all dance around these same mysterious underlying causes. I hope the resulting datasets can be used for a more predictive approach as they are broadened, but as the authors caution, generalizing the conclusions may be difficult as each mutant needs to be considered on a case-by-case basis.

This is a report to suggest that changes in physiological concentration of metabolites also change cellular proteostasis. We show that there is an alteration of proteostasis upon changing metabolism that is independent of the canonical chaperones. Thus, the focus of the work was to test if metabolites can alter proteostasis in vivo and change mutational buffering.

The reviewers have made an important point here - can we predict what mutations (in which

proteins) will be buffered if we change the metabolism in a certain way? We would require a more exhaustive dataset with multiple protein mutant libraries and with many different metabolic changes (that are quantified along with transcriptome) to develop a dataset that can be used for training machine learning models to predict the effect of metabolites in a mutant specific manner. We have already reported this complication exhaustively in (Dandage et al., 2015) and later in (Dandage et al., 2018). The small molecules have a complex effect depending on the protein fold and the mutations. Collecting this sort of dataset is complicated and involves substantial effort. The work is ongoing and the current one is first in this series where we start generating the datasets required for this type of analysis.

We agree, it is a LOT of work to make and test protein mutant libraries. If the authors do not

wish to do the experiments to get a more exhaustive dataset with multiple protein mutant libraries, they should back off some of their strong claims that this is so generalizable and scale back their statements to be specific to the system & proteins they examined. Perhaps the discussion section would be a better place for the authors to discuss the broader implications of what mutational buffering at the level of these two proteins means.

Ans: We have very specifically mentioned in the Results section that the buffering is specific for sensors and sometimes they are protein-specific. We have highlighted these as comments in the word document and have taken care not to overgeneralize the statement.

Specific points/questions:

1. Better defining “mutational buffering” up front is needed. This term can be used very broadly and differently depending on context.

We have tried to define this in the introduction and **Figure 1**. We have also streamlined the introduction to make this concept clearer.

Figure 1 makes this much more clear and is a valuable addition to the manuscript. note for

Ans: Noted.

2. Addressing how chemical chaperones might have differing effects on different protein folding states may be useful in light of what we know about the protein composition (i.e. how

introducing salt will affect exposed hydrophobic sites, how hydrophobic in general are the proteins they looked at, etc.)

Suggestion duly taken and now we have added a paragraph in the implications section highlighting the same. Additionally, we have added structural analysis of the mutants (please

see a response below) to indicate the type of mutations that seem to be buffered by altered metabolite composition.

The implications paragraph covers this point sufficiently.

Ans: Thank you.

3. Authors should address how their result that molecular chaperones do not affect folding of

isolated mutants since that is relatively unexpected. Is this perhaps because the proteins

studied were non-native to their e. coli strain?

While there are recent reports showing that not all mutations are dependent upon chaperones

for folding(Tripathi et al., 2016), it is a common knowledge that overexpression of specific chaperones do not help folding of all proteins (or mutants). We have now included a section showing how the GFP model is able to detect alteration in canonical proteostasis machinery (line 300-310, **Figure 5H, S4E**). There are two important points we would like to clarify. First, from the library of mutants, few were isolated that showed different fluorescence (folding) in two different strains. These mutants were not chaperone dependent, although the library had other mutants that were chaperone dependent. Second, not all mutants are chaperone dependent. We still do not understand what types of mutations (in a protein specific manner) leads to dependence on which chaperone. We have isolated GroEL/ES dependent GFP mutants in a collaborative study using the same mutant library.

One reason we find the paper confusing is that they really are referring to [general] mutational

buffering as two things: 1 - due to molecular chaperon assistance and 2 - through metabolite dependent changes on protein folding. Yet they include statements like this one in the abstract

throughout the paper:

“

Since proteostasis can modulate mutational buffering, if change in metabolism has the ability to change proteostasis, arguably, it should also alter mutational buffering. Building on this, we find that altered cellular metabolic states in *E. coli* buffer distinct mutations. Buffered-mutants had folding problems *in vivo* and were differently chaperoned in different metabolic states

“

where they seem to be mixing the two mutational buffering mechanisms or leaning too heavily

on the assistance from molecular chaperones to explain changes that seem to be due to metabolite dependent changes Again, we think that the authors’ are trying to say something very specific about 1) proteostasis through molecular chaperones 2) mutational buffering through metabolites independently and 3) how metabolites/osmolytes affect chaperones. However, we do not in general feel that they were very clear or specific in their language throughout the paper in clarifying what exactly they meant. For example, many times in the paper the authors use “proteostasis”, which we associate with molecular chaperones. However, it is clear from their language that they are using mutational buffering and proteostasis interchangeably at this point

Ans: Proteostasis is defined as the sum of all the mechanisms that affect protein biogenesis, folding, targeting, and degradation. Our idea was to use this definition without limiting it to molecular chaperones as we find that small molecules indeed affect protein folding in the cell. However, we realize the confusion and now have changed the word “proteostasis” with “ cellular protein folding capacity”.

4. This is related to the idea of a stimulatory role for metabolites on chaperone action (a broader indirect effect) rather than a specific role for metabolites stabilizing certain mutants?

This is one possibility however; the other possibility is that metabolites (like osmolytes) alter solvent structure or bind to (un)structured regions to help folding. In case metabolites only potentiated the function of molecular chaperones we would have seen chaperone dependent

mutants to be hyperactive in presence of these metabolites (upon altering metabolism - WG350(S)). This is not the case (the mutants are not found to be dependent on chaperones), the chaperone-dependent mutants are not altered, but mutants that are altered are inherently

dependent on the small molecules. We believe this is indeed due to the ability of the small molecules to act on the proteins (solvent) themselves. It is unexpected because there have been no reports of metabolites being a part of the proteostasis network and its capacity to function independently. As explained in the introduction taking an endogenous protein as a sensor would restrict our capacity to uncouple effect on protein folding from other compensatory effects. However, not all mutants in the two libraries used in the study are independent of molecular chaperones (sGFP from the same library is a substrate for GroEL). We do not find stimulatory role of metabolite on molecular chaperones in buffering of these mutants as these mutants are not assisted by GroEL/ES or dnaK/J/GrpE machinery both *in vivo* and *in vitro* as shown in **Figure 5D, 5F**.

I think the points made in the first paragraph of this response are a good indication that it is not a broader indirect effect. We agree.

Ans: Thank you

5.This study goes deep into two strains, esp. with the metabolomics and transcriptomics, but

chemical perturbations that have effects on osmolarity could also be used.

A. We agree and this is what initiated us to use NaCl in the later part of the work. However,

As the starting point we did not want to alter osmolarity directly or abruptly as it has been shown earlier that changes in osmolarity leads to alteration in a large number of proteostasis pathways including translation, folding and degradation. In a separate section of the manuscript (**Figure 7**) we have used chemical perturbations that cause large osmolarity changes (400mM Sucrose, 400mm Sorbitol to CSH4 strain) and did not observe buffering. This suggested that osmotic shock alone is not altering the buffering in CSH4 but the cellular response to osmotic shock (the final cellular milieu) determined folding capacity of these cells when probed with the mutants we isolated.

*B.* We did use NaCl from outside in the strains chosen but the concentration was so chosen that it did not affect the growth of the organisms significantly. Transcriptomics and metabolomics of both the strains under physiological conditions of growth and with mild osmotic shock were recorded and analyzed (This section is now expanded as suggested). We made sure using these that the canonical pathways of proteostasis are not significantly altered. This assured that we could distinguish the effect of metabolites and chaperones on folding *in vivo.*

C. We did not use other chemicals that induce a strong osmotic shock as this would convolute the analysis of the mutants that are buffered. It would be difficult to resolve if the molecules used for the treatment (present at non-physiological concentrations) led to buffering (this was done in our previous work (Bandyopadhyay et al., 2012)), or if the cellular response to the osmotic shock led to the difference in folding observed.

D. Instead, we used an array of small molecules at moderately high concentrations to modify the cellular metabolism (**Figure 7**) and check if these molecules can affect folding.

The authors’ response has sufficiently addressed comment #5. Figure 7 shows the results of the KCL, sucrose, and sorbitol supplementation and Figure S1B shows the growth results with NaCl addition.

Ans: Noted

6. The evolved strain in high salt somewhat addresses this, but have the authors speculated or done any experiments on the effect of more transient or acute challenges and mutational buffering using these tools and libraries? (We are obviously interested in this as it is making us rethink some of our own chemical perturbations in deep mutational scans of ubiquitin)

We have done some preliminary experiments with acute stress (data shown below) which is similar to what we obtained with chronic stress. However, with the current reporter (mutant GFP/Wt mCherry) it is not possible to follow an acute response in real time for the different strains. We are also interested in understanding the difference between acute and chronic alterations in metabolism (or stress response), the development of a suitable tool to study this

however is outside the scope of this current work.

**Figure:** Plot for gel-based quantification of intact GFP (left) and representative blots (right) to

chase degradation of C1 GFP in presence and absence of acute osmotic stress (15min) to

CSH4 and WG350 followed by protein induction for 5 min and translation arrest by

Chloramphenicol. GFP intact is shown by immunoblotting with anti-GFP antibody.

We agree it is outside the scope of the current work, and their response is fine. We think we

were more curious on this point than demanding they address it in this paper.

Ans: Noted

7. The initial mutant the authors found in the Gm-R library were both to glycine and both in the

same sequence space, and it is unclear what the WT residues are.

We have now included the original residues in the naming convention. The reference has been

provided for Gm-R in the results however, we will include the complete sequence in supplementary or bio project if required. The mutant library used were glycine-duplet substitution library as shown schematically in **Figure S1A**

**I think the complete sequence would be a good addition to the supplement. Figure S1A**

**makes the library more clear and is a good addition.**

Ans: We are glad that the reviewer has liked the schema. We have added the sequence and alignment in Figure S1B and S2C.

8. The claim the authors make that they are less active in the transporter-deleted metabolic state may be a stretch. It would be nice to see where on the protein structure these mutants are – biophysically they might have a simpler explanation for their decrease in protein activity that is independent of the metabolites and similarly for their enhanced activity in the same strain with the addition of salt (i.e. may be restoring the charge disrupted by a glycine rather than mutational buffering).

If a mutant protein is less active than WT, it may have nothing to with proteostasis but if they are differently active in two different strains, it may be either due to difference in protein amount

(proteostasis) or due to difference in specific activity. With GFP we confirm that these mutants

do not have differences in specific activity. We demonstrate that this is due to different steady

state levels of mutant proteins in the different strains by using GFP (*in vivo* quantitation/biophysics). We show that it is dependent on cellular proteostasis by *in vivo* degradation experiments. Additionally, we provide evidence that these mutants are most probably destabilized mutants and not inactive ones with detailed structural analysis (now added in **Figure 2F, 2H (Gm-R) and 3H, 3I (GFP)**). This is also supported by the *in vitro* refolding experiments in presence and absence of chemical chaperones like TMAO that aid folding of some of these mutants. We had not included a comprehensive structural analysis of

the mutants as we had published it earlier(Bandyopadhyay et al., 2012; Dandage et al., 2015). We have now included some analysis of the mutants to support our claims related to protein folding. Most of these mutants are predicted to affect protein stability, while majority do not lead to any charge alterations ruling out simple effect of salt on stability. We have also shown that NaCl directly does not increase folding of the mutants *in vitro*. The mutants GG1(I26G) and GG2(I27G, R28G) of Gm-R shows difference in activity between CSH4 and WG350 in absence of salt. So, the difference in their activity is not salt-dependent *in vivo*.

While we still think some structural analysis would help, the conservation plots in figures 2 & 3 start to tell us we were asking for in this comment.

Ans: The Figures are all the structural informations obtainable (depth, ASA, ΔΔG). Secondary structural analysis, B-factor analysis, phi-psi analysis, were not reported as had no correlation with buffering (we had shown part of this in our previous paper – Bandyopadhyay et al). So we had already provided a comprehensive analysis of the structure that provide information leaving out the other correlates that did not provide sufficient information.

9. The implications section could be grounded in a broader context tying together several threads in a proteostasis context rather than the larger leap to aging and metabolism.

Eg. ATP levels as a protein stability/oligomerization agent: https://www.ncbi.nlm.nih.gov/pubmed/28522535, other classic literature on metabolic products and stability: https://www.ncbi.nlm.nih.gov/pmc/articles/PMC3996911/ , and amino acids as a refolding agent: <https://www.ncbi.nlm.nih.gov/pubmed/15458311> The implication section is now rewritten based on the suggestion.

I think the re-written implication section is good.

Ans: Thank you.

We review non-anonymously, Erin Thompson and James Fraser, UCSF, and have posted our

comments on the BioRxiv preprint as well.

We thank the reviewer for his help in understanding certain questions through BiorXiv

**Reviewer comments, third version:**

REVIEWERS' COMMENTS:

Reviewer #1 (Remarks to the Author):

We have no further comments

**Author rebuttal, third version:**
